# Broad-Spectrum Naphthyl-Substituted Diaminoquinolines Inhibiting the AdeG Efflux Pump of *Acinetobacter baumannii*

**DOI:** 10.1021/acsinfecdis.5c00722

**Published:** 2026-01-05

**Authors:** Rushikesh Tambat, Aysegul Saral Sariyer, Emrah Sariyer, Marcela Olvera, Mithila Farjana, Napoleon D’Cunha, John K. Walker, Helen I. Zgurskaya

**Affiliations:** † Department of Chemistry and Biochemistry, 6187 University of Oklahoma, Norman, Oklahoma 73019, United States; ‡ Department of Nutrition and Dietetics, Faculty of Health Sciences, Artvin Coruh University, 08000 Artvin, Turkey; § Vocational School of Health Services, Medical Laboratory Techniques, Artvin Coruh University, 08000 Artvin, Turkey; ∥ 12274 Saint Louis University School of Medicine, St. Louis, Missouri 63110, United States; ⊥ Department of Chemistry, Saint Louis University, St. Louis, Missouri 63110, United States

**Keywords:** *Acinetobacter baumannii*, multidrug efflux pumps, efflux pump inhibitors

## Abstract

AdeFGH and AdeIJK, the two homologous multidrug efflux pumps of the resistance-nodulation-division superfamily of transporters, play distinct roles in *Acinetobacter baumannii* physiology and antibiotic resistance. Unlike ubiquitous AdeIJK, AdeFGH is strain-specific, typically expressed at low levels, and if overproduced, it enables resistance to a narrow spectrum of antibiotics, e.g., fluoroquinolones or chloramphenicol. In this study, we report that representatives of naphthyl-substituted diaminoquinolines targeting AdeIJK are also active against AdeFGH. We isolated AdeFGH-overproducing strains from the clinical AYE and Ab5075 isolates lacking AdeIJK and AdeABC pumps and demonstrated that these inhibitors are active in *A. baumannii* strains with different genetic backgrounds. The inhibitors potentiate the antibacterial activities of various antibiotics and enhance the bactericidal properties of the fluoroquinolones. We further analyzed how amino acid substitutions in the substrate translocation tunnels of AdeG affect the efflux properties of this pump and its sensitivity to inhibitors and compared them to the analogous substitutions in AdeJ. Our results suggest that the inhibitors engage similar contacts within the deep binding pockets of the two pumps but differ in their interactions in the entrance and the proximal binding sites. We conclude that the broad-spectrum activities of the diaminoquinolines as well as other inhibitors likely arise from the interactions within the deep-binding pockets, but their specificity is determined in the proximal-binding sites of the pumps.

## Graphical abstract

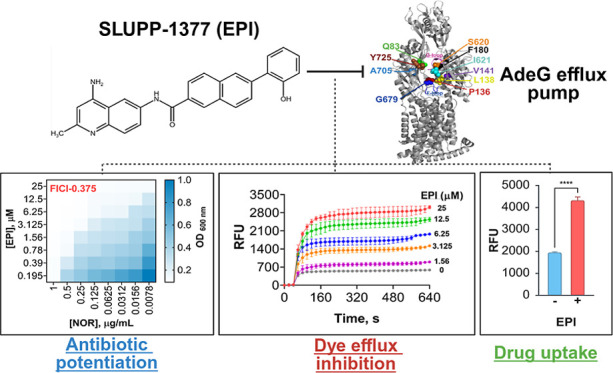

Resistance-nodulation-cell division (RND)-family efflux pumps play an important role in the antibiotic resistance of Gram-negative bacterial strains.^1^ To date, six efflux pumps belonging to the RND family, AdeFGH, AdeIJK, AdeABC, CzcABCD, AbeD, and ArpAB, have been identified in *Acinetobacter baumannii*. Except for CzcABCD, which is a metal ion efflux transporter, all other RND pumps can provide resistance to various antibiotics.^2^ This study is focused on the AdeFGH efflux pump, which was initially identified using AdeABC and AdeIJK-deficient *A. baumannii* strains^3^ and was found to confer resistance to chloramphenicol (CHL), trimethoprim (TMP), ciprofloxacin, and clindamycin (CLI).^1^ Also, at least in some strains, the inactivation of *adeFGH* renders cells more susceptible to CHL, imipenem, and doxycycline.^4^ The AdeFGH pump is not typically expressed in wild-type (WT) strains under laboratory conditions.^5^ However, exposure to antibiotics can select mutations in the promoter region of AdeFGH and the LysR-type transcriptional regulator named AdeL, leading to the overexpression of the pump.^3,6^ Overexpression of AdeFGH has a higher fitness cost to the cell than overexpression of the AdeABC and AdeIJK efflux pumps.^7^ Among substrates of the AdeFGH efflux pump are various antibiotics and possibly autoinducing molecules synthesized during biofilm formation.^8^ The substrate specificity and physiological properties of AdeFGH are similar to those of its better characterized homologues such as MexEF-OprN of *Pseudomonas aeruginosa* and BpeEF-OprC of *Burkholderia thailandensis*. Because of its role in antibiotic resistance and biofilm formation, the AdeFGH efflux pump could be considered as a potential antibacterial target and the development of bacterial efflux pump inhibitors could aid in studies of this pump and in the development of therapeutics against antibiotic-resistant *A. baumannii* strains.

Like other tripartite RND efflux pumps from Gram-negative bacteria, AdeFGH comprises a periplasmic membrane fusion protein AdeF, an inner membrane RND transporter AdeG, and an outer membrane channel AdeH.^3^ The cryo-EM structure of AdeG was recently reported and, similar to homologous transporters, was found to contain the transmembrane domain, the porter domain, and the docking domain.^9^ The transmembrane domain of AdeG is made up of 12 α-helices per monomer that embed the protein into the inner membrane, and this region also contains the amino acid residues needed for proton translocation. The porter domain is the region of the transporter that contains the two substrate-binding sites, named the proximal and distal binding pockets. These two binding pockets are separated by a G-loop, which is essential for substrate binding and transport.^10^ The docking domain forms a funnel shape that helps move the pump substrates further into the efflux pump complex.^11^ The transport mechanism involves the RND transporter trimer cycling through three conformational states: access (or loose) state, binding (or tight) state, or extrusion (open) state.^9,10,12^ The protomers cycle through these three states to (1) allow pump substrates to enter the transporter in the access state, (2) move substrates further into the drug-binding pocket during the binding state, and (3) funnel substrates into the channel created by the periplasmic and outer membrane components in the extrusion state. The conformational transitions of transporter protomers are driven by changes in the protonation of specific amino acid residues located in the transmembrane domain.^13^

Previous studies identified several synthetic or natural efflux pump inhibitors acting on major RND pumps, e.g., *Escherichia coli* AcrAB-TolC^14^ and *A. baumannii* AdeIJK,^15,16^ but efforts to identify inhibitor candidates effective against AdeFGH are limited. Phenylalanine–arginine β-naphthylamide (PAβN), one of the most extensively studied synthetic efflux pump inhibitors in *A. baumannii*, has been shown to inhibit the AdeFGH pump by lowering the minimum inhibitory concentrations (MICs) of TMP, CHL, and CLI.^17^ In addition, a series of quinoline-based compounds were designed and analyzed by experimental and molecular modeling for inhibitor discovery against AdeG. As a result, two compounds were found to potentiate CHL more than 16-fold (from 512 μg/mL to 32 μg/mL) in Ab5075-CHL, an AdeG-overexpressing strain.^9^

We recently disclosed inhibitors specific to the AdeIJK pump.^18^ While counter screening for AdeFGH, we identified naphthyl-substituted diaminoquinolines (DAQs) **SLUPP-1377** and **SLUPP-1021** that inhibit not only AdeIJK but also the AdeFGH efflux pump. In this study, we investigated the mechanism of these broad-spectrum inhibitors acting on AdeFGH by constructing and characterizing single amino acid substitutions in the ligand translocation path of AdeG and by comparing the action of the inhibitors on AdeIJK and AdeFGH pumps.

## Results

### Overproduction of AdeFGH Is Selected in the Absence of AdeIJK and AdeABC

*A. baumannii* AYE and Ab5075 strains are the two best characterized clinical multidrug resistant (MDR) isolates that overproduce, albeit to different extents, the two major RND pumps AdeIJK and AdeABC.^19^ These strains also contain target- and modifying enzyme-mediated antibiotic resistance. We previously reported that the double-compromised derivatives AYE Δ2 (Δ*adeB* Δ*adeIJK*) and Ab5075 Δ2 (*adeB*::T26 Δ*adeIJK*), lacking activities of both these pumps, were hypersusceptible to various antibiotics.^19^ These efflux-deficient strains were found to overproduce several alternative efflux pumps but not AdeFGH, the expression of which remained at very low levels. To select mutants overproducing AdeFGH, the efflux-deficient AYE Δ2 and Ab5075 Δ2 cells were exposed to a 1 × −8 × MIC of CHL, a known substrate of AdeFGH. A few single-step CHL-resistant (CHL^r^) colonies were isolated, and their *adeG* and *adeL* genes were PCR amplified and sequenced. One isolate of AYE Δ2 containing the AdeL^T319K^ variant and two Ab5075 Δ2 derivatives containing AdeL^V139G^ and AdeL^IS5^ mutations were selected for further studies.

The three CHL^r^ mutants were found to overproduce AdeG, as seen from the immunoblotting analysis of lysates prepared from the respective cells (Figure 1A, Table S1). We next measured the MICs of representative antibiotics in AYE and Ab5075 strains and their derivatives and compared them to the MICs of antibiotics in the antibiotic-susceptible *A. baumannii* ATCC17978 strain, its efflux-deficient derivative AbΔ3 (Δ*adeIJK* Δ*adeAB* Δ*adeFGH*), and the complemented AbΔ3­(pAdeFGH) overproducing a plasmid-borne AdeFGH (Table 1).^20^ In agreement with previous data,^19^ AYE and Ab5075 were more resistant than ATCC17978 to all tested antibiotics (Table 1). Inactivation of the major Ade efflux pumps sensitized all three strains to antibiotics, although the MICs of azithromycin (AZM) and gentamicin (GEN), the substrates of AdeABC, were affected only in the AYE and Ab5075 backgrounds. The CHL^r^ isolates as well as AbΔ3­(pAdeFGH) were resistant to CHL, norfloxacin (NOR), and nalidixic acid (NAL), the substrates of AdeFGH, but not to AZM and GEN (Table 1). Thus, overproduction of AdeFGH in different genetic backgrounds leads to similar changes in the susceptibilities of *A. baumannii* to antibiotics.

**Figure 1:**
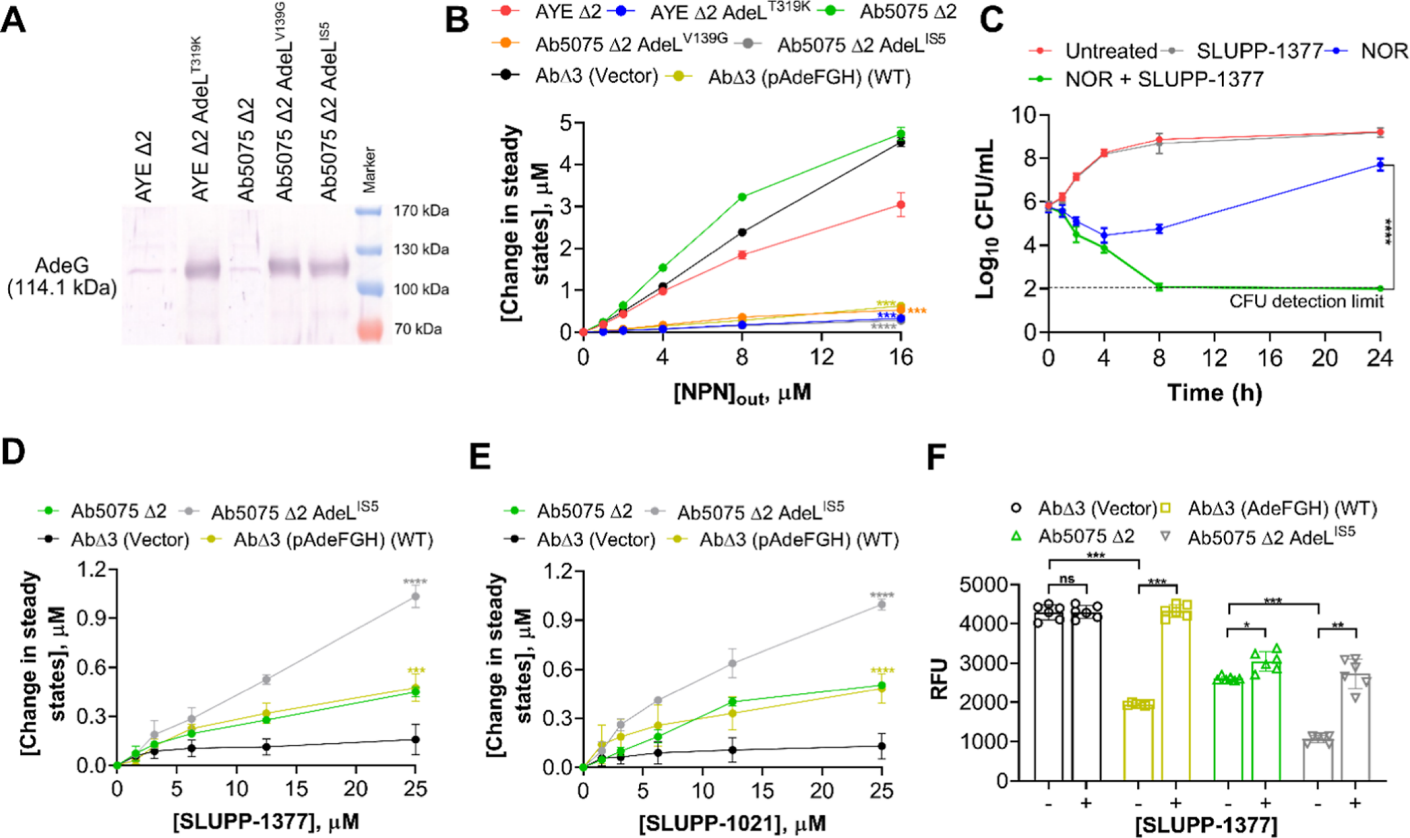
(A) Immunoblotting analysis of cell lysates from *A. baumannii* AYE Δ2 and Ab5075 Δ2 cells and their mutated AdeL variants. Expression of AdeG (∼114.1 kDa) efflux protein was visualized with an anti-AdeG polyclonal antibody. (B) Steady-state NPN accumulation levels calculated from the kinetic curves shown in Figure S1. NPN was used at the final external concentration ranging from 0 μM to 16 μM. (C) Time-kill curves of Ab5075Δ2 AdeL^IS5^ show the bactericidal effect of SLUPP-1377 (3.125 μM) + NOR (128 μg/mL) combination. (D,E) Steady-state NPN (8 μM external concentration) accumulation levels calculated from the kinetic curves shown in Figure S2 (SLUPP-1377) and Figure S3 (SLUPP-1021). The EPIs were used at a concentration ranging from 0 μM-25 μM. Each data point represents the average of two biological replicates with two technical repeats ±standard deviation (SD). (F) Accumulation of NOR in the absence (−) and presence (+) of SLUPP-1377 (25 μM). Relative fluorescence units (RFU) were calculated after normalizing the background fluorescence of NOR to untreated bacterial cell-free supernatants. Each data point represents the average of two biological replicates with three technical repeats ±SD. Data was analyzed by a *t*-test. Values were considered statistically significant at *P < 0.05 and highly significant at **P < 0.01, ***P < 0.001, and ****P < 0.0001. ^#^ns, nonsignificant.

**Table 1:** MICs of Antibiotics in *A. baumannii* AYE, Ab5075, and ATCC17978 Strains and Their Indicated Efflux-Pump Deletion and Overexpressing Variants^a^

	MICs (μg/mL)				
strains	CHL	NOR	NAL	AZM	GEN
AYE	256	256	>1024	32	1024
AYE Δ2	64	16	64	0.125	32
AYE Δ2 AdeL^T319K^	512	256	512	0.25	32
Ab5075	128	256	>1024	16	512
Ab5075 Δ2	32	8	64	0.125	64
Ab5075 Δ2 AdeL^V139G^	256	128	256	0.125	64
Ab5075 Δ2 AdeL^IS5^	512	128	512	0.25	64
ATCC17978	64	4	16	2	16
Δ3 (Vector)	8	0.25	2	0.5	8
Δ3 (pAdeFGH) (WT)	32	1	16	0.5	8
Δ3 (pAdeIJK)	32	4	16	0.5	16

a Abbreviations—CHL, chloramphenicol; NOR, norfloxacin; NAL, nalidixic acid; AZM, azithromycin; GEN, gentamicin.

We also analyzed the activity of the overproduced AdeFGH in the bacterial-killing-independent efflux assay using a fluorescent membrane probe 1-*N*-phenylnaphthylamine (NPN). NPN is a 219 Da small molecule that cannot cross the outer membrane but is highly fluorescent when bound to phospholipids.^21^ In these experiments, NPN is added to AdeFGH-deficient and overproducing cells at final concentrations ranging from 0 to 16 μM, and the increase in NPN fluorescence is monitored in real time (Figure S1). In agreement with NPN being a substrate of AdeFGH,^20^ in all tested genetic backgrounds, cells overproducing the AdeFGH pump had lower intracellular NPN accumulation levels compared to the efflux-deficient cells (Figure 1B).

### Naphthyl-Substituted DAQs Potentiate the Activities of Antibiotics in the AdeFGH-Overproducing Cells

We next screened the previously disclosed efflux pump inhibitors (EPIs)^18^ from the DAQ class of compounds for potentiation of CHL in AdeFGH-overproducing cells. Among tested compounds, **SLUPP-1377** and **SLUPP-1021** were identified as EPIs targeting AdeFGH and were further characterized in both bacterial-growth-dependent and independent assays. The two compounds had modest antibacterial activity in efflux-deficient AYE Δ2, Ab5075 Δ2, and AbΔ3 cells with MICs ranging between 6.25 and 25 μM. The overproduction of AdeFGH (Table 2) or AdeIJK (Table S2) increased these MICs to 100 μM–200 μM, suggesting that both EPIs are substrates of the efflux pumps and likely follow the same translocation path as antibiotics. To characterize the inhibitory activities of DAQs, we determined their minimal potentiating concentrations, which decrease the MIC of an antibiotic by 4-fold (MPC_4_). Both **SLUPP-1377** and **SLUPP-1021** were found to potentiate the antibacterial activity of CHL, NOR, and NAL against cells overproducing AdeFGH in different genetic backgrounds (Table 2). In this experiment, we expect that inhibitors will not potentiate the activities of antibiotics in cells lacking the target pump and will be more effective when the target is available. However, the sensitivity to the inhibitory action of the compounds varied between the different strains. The lowest MPC_4_ values of 1.56 μM–3.125 μM for **SLUPP-1377** and 6.25 μM for **SLUPP-1021** were found for the efflux-deficient AYE Δ2 and Ab5075 Δ2 cells producing the low levels of AdeFGH (Figure 1A). These MPC_4_ values increased by 2–4-fold in the CHL^r^ mutants (Table 2). This result suggests that potentiation is sensitive to the levels of AdeFGH expression. Indeed, no potentiation of antibiotics was found in AbΔ3 cells lacking all three Ade pumps (MPC_4_ = MIC), whereas strong potentiation is seen in the AbΔ3­(pAdeFGH) cells carrying a plasmid-borne AdeFGH. **SLUPP-1377** and **SLUPP-1021** display synergy (FICI ≤0.5) with antibiotics in cells producing either low or high levels of AdeFGH, whereas indifference (FICI >1 - ≤ 4) was observed in cells lacking efflux pumps (Table S3).

**Table 2:**
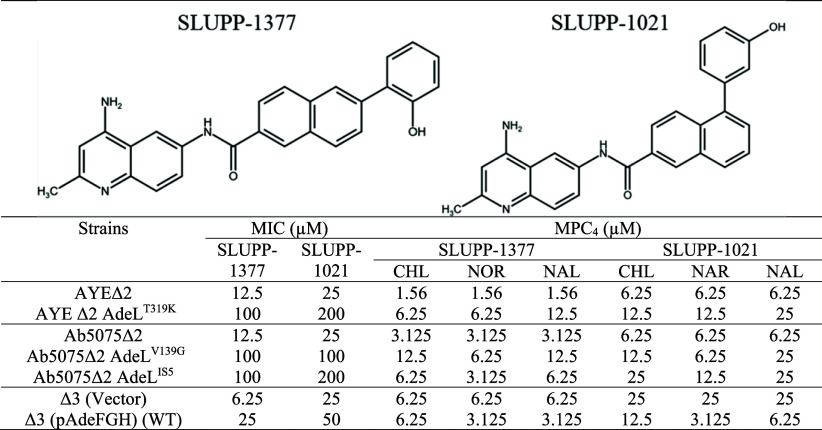
MPC_4_ of Naphthyl-Substituted DAQs with Substrate Antibiotics in Efflux-Deficient *A. baumannii* AYE Δ2, Ab5075 Δ2, and ATCC17978 Δ3 and Their Corresponding AdeG-Overproducing Variants

Next, the time-kill experiments on Ab5075 Δ2 AdeL^IS5^ were performed to determine whether the NOR (1 × MIC) plus **SLUPP-1377** (MPC_4_) combination is bactericidal. As expected, **SLUPP-1377** alone (subinhibitory concentration) behaved similarly to the untreated control and exhibited no antibacterial effect. NOR alone initially showed a reduction in the number of colony-forming units (CFU) up to 4 h, but regrowth was observed afterward. However, we noticed that the combination of NOR with **SLUPP-1377** killed the bacteria in 8 h up to the detection limit with no regrowth up to 24 h of incubation. A ≥ 2-log_10_ decrease in CFU/mL was observed between the combination and NOR after 24 h, and the number of surviving organisms in the presence of the combination was ≥2 log_10_ CFU/ml below the starting inoculum, representing synergy (Figure 1C). Thus, the combination of **SLUPP-1377** with NOR is more efficient in the killing of *A. baumannii* cells than NOR alone.

In agreement with previous results,^18^ both **SLUPP-1377** and **SLUPP-1021** compounds were found to be cytotoxic to A549 cells (human alveolar basal epithelial cells) with cytotoxic concentrations CC_50_ values of 7.6 μM for **SLUPP-1377** and 38.1 μM for **SLUPP-1021.** Although these compounds are not suitable for animal studies, they offer a tool to gain insight into the mechanisms of efflux by AdeFGH and its inhibition.

### DAQs Increase Intracellular Accumulation of Fluorescent Probes and Antibiotics, the Substrates of AdeFGH

Overexpression of efflux pumps is expected to reduce the intracellular concentration of antibiotics, and their inhibition prevents the extrusion of antibiotics to restore their antimicrobial activity. To confirm that the DAQs-mediated inhibition of efflux leads to increased intracellular accumulation of AdeFGH substrates, we first analyzed whether these inhibitors increase the level of intracellular accumulation of fluorescent probes. In these experiments, we used the fluorescent membrane probe NPN, the concentration of which was set to 8 μM, and the inhibitors were added in final concentrations ranging from 0 μM to 25 μM. In the presence of the inhibitor, AdeFGH-overproducing cells accumulated up to four times higher amounts of NPN as seen in the corresponding increase in NPN fluorescence (Figures 1D,E and S2–S4), but the levels of NPN accumulation varied depending on the strain. In contrast, no changes in NPN accumulation in the presence of inhibitors were seen in the efflux-deficient cells.

We next analyzed intracellular accumulation of the NOR antibiotic in AdeFGH-deficient and overproducing cells. We found that efflux-deficient cells have accumulated statistically higher levels of NOR, whereas overproduction of AdeFGH reduced this intracellular uptake (Figure 1F). When AdeFGH-overproducing cells were treated with **SLUPP-1377** (25 μM final concentration), the intracellular accumulation of NOR significantly increased. As expected, no significant change in the NOR uptake was observed in cells lacking efflux pumps after treatment with **SLUPP-1377** (Figure 1F). Thus, **SLUPP-1377** (Figure 1D,F) and **SLUPP-1021** (Figure 1E) inhibit the efflux activity of the AdeFGH pump, albeit their efficiency is dependent on the genetic background of *A. baumannii* strains.

### Substitutions in AdeG Binding Sites Modify Efflux Efficiency

To establish whether DAQs act by the same mechanism of inhibition against AdeFGH and AdeIJK, we constructed ten mutants with single amino acid substitutions in the putative substrate translocation path of AdeG (Table 3), in positions analogous to those constructed previously in AdeJ.^22^ Structural studies suggested that the ligand binding patterns between AdeG, AdeJ, and AdeB efflux pumps are similar, and the residues involved in ligand binding within AdeG exhibit a high degree of similarity to those found in the homologous efflux pumps.^9^ Therefore, we selected residues that are predicted to be involved in ligand translocation based on the cryo-EM structure of AdeG^9^ and were previously found to contribute to substrate and inhibitor recognition in the AdeJ pump.^22^ The substitution G679I is in the F-loop of AdeG, which separates the drug entrance and the Proximal Binding Pocket (PBP) and contributes to substrate specificity of Ade and homologous pumps.^23,24^ The G-loop S620A and I621A substitutions are located at the interface separating the PBP from the Distal Binding Pocket (DBP) and possibly participate in transferring the substrate along the translocation path.^9,25^ The three other substitutions Y725A, A705I, and Q83A were introduced into the PBP, whereas substitutions P136A, L138A, V141C, and F180C are located in the DBP (Figure 2A).

**Table 3:** MICs of Substrate Antibiotics in the Efflux-Deficient *A. baumannii* ATCC17978 Strain Carrying either an Empty Vector (Vector) or Producing the WT and the Indicated AdeG Variants

		MICs (μg/mL)		
strains	location	CHL	NOR	NAL
vector		8	0.25	2
WT		32	1	16
G679I	F-loop	**8**	**0.25** ^a^	**4**
Q83A	PBP	32	1	16
A705I		32	2	16
Y725A		16	0.5	8
S620A	G-loop	16	1	16
I621A		16	**0.25**	8
P136A	DBP	16	**0.25**	**4**
L138A		32	1	16
V141C		32	1	16
F180C		**8**	**0.25**	**4**

a Here and in Tables 4 and 5, values that differ from the WT by four or more folds are shown in bold.

**Figure 2:**
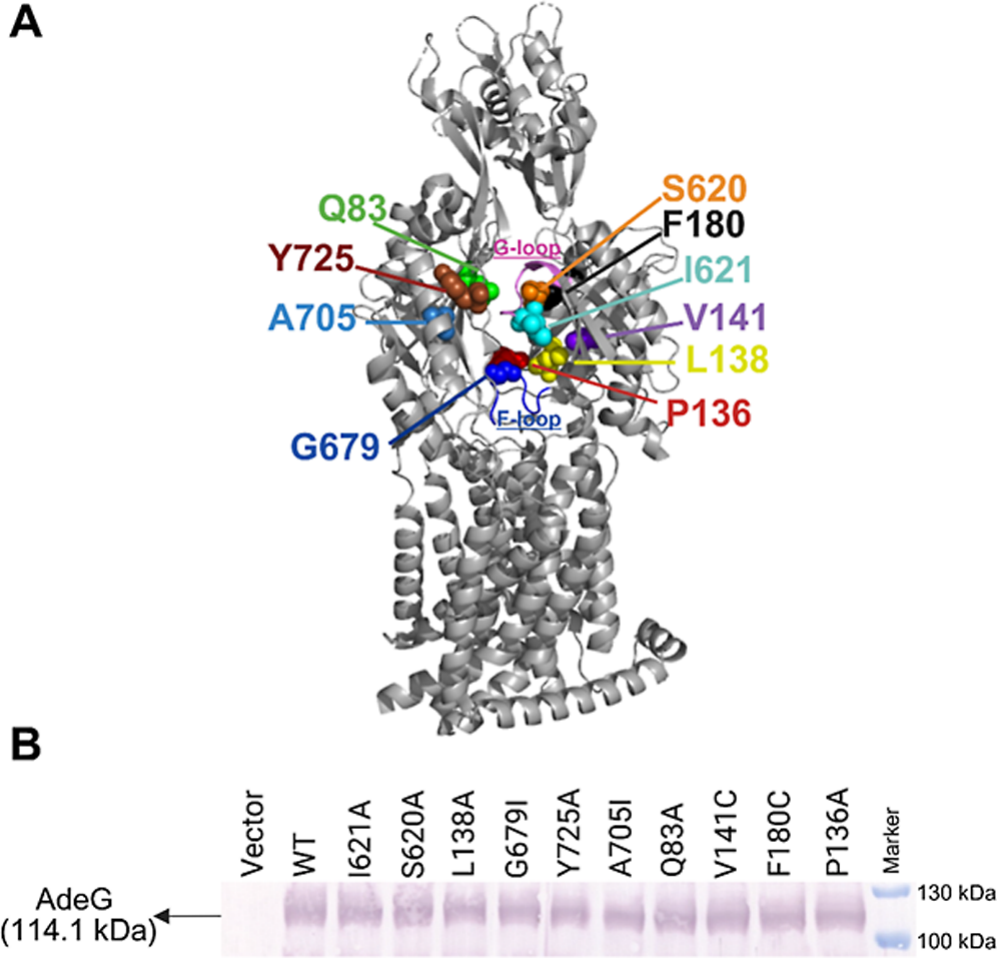
(A) The monomeric subunit of AdeG (side view). The amino acid residues in the substrate translocation path used in this study for substitution are highlighted with spheres in different colors (PDB ID: 8YR0).^9^ The F-loop (Residues 675–684) and G-loop (Residues 618–629) are colored blue and magenta, respectively. (B) Immunoblotting analysis of cell lysates from *A. baumannii* Δ3-pore cells carrying an empty vector, WT (AdeFGH) pump, and indicated AdeG single amino acid-substituted variants. AdeG (∼114.1 kDa) variants were visualized with an anti-AdeG polyclonal antibody.

To analyze functional activities of AdeG variants, the WT and mutated *adeG* genes were integrated into the Tn7 site of the chromosomes of AbΔ3 or expressed from the plasmids in its hyperporinated AbΔ3-Pore derivative, producing a large pore in the outer membrane. The expression of AdeFGH was induced by growing cell cultures in the presence of 1% l-arabinose. Immunoblotting analyses showed that all AdeG variants were expressed at levels comparable to those of the WT protein in both AbΔ3 (Figure 2B) and its hyperporinated AbΔ3-Pore derivative (Figure S5; Table S4).

We next measured MICs of CHL, NOR, and NAL in cells producing AdeG variants (Table 3). The antibiotic susceptibility assays showed the AdeG mutants G679I and F180C were associated with a complete loss of antibiotic efflux, as seen from a 4- to 8- fold decrease in MICs in AbΔ3 producing these AdeG variants. The AdeG mutants I621A and P136A showed partial loss of activity, which is 4-fold for NOR and 2-fold for CHL and NAL. Cells producing the remaining AdeG variants demonstrated antibiotic susceptibilities similar to the WT AdeFGH, with 2-fold variability at most. The MIC results remained within the 2-fold differences in the hyperporinated AbΔ3-Pore cells (Table S5), suggesting that the outer membrane of these cells does not alter the MIC values of these antibiotics significantly.

Next, the kinetics of efflux by the AdeG mutants of NPN (Figures 3A,B and S6) and another fluorescent DNA-binding probe, ethidium bromide (EtBr) (Figure S7), were analyzed. In agreement with MIC measurements, we found that AdeG mutants F180C and G679I were the least effective in preventing the intracellular accumulation of both NPN and EtBr and were similar to the negative-efflux-deficient AbΔ3-Pore control (Figures 3A and S7A). Thus, these two mutant variants are significantly impaired in their ability to efflux fluorescent probes and antibiotics. AdeG mutants I621A, P136A, and Y725A were also impaired in efflux activities but with some substrate specificity. The accumulation of NPN in cells producing AdeG mutants I621A, P136A, and Y725A was intermediate between the positive and negative controls, whereas these mutants were completely defective in reducing the accumulation of EtBr (Figures 3A and S7A). The AdeG mutants V141A and S620A showed NPN uptake profiles most similar to the WT, with the mutants Q83A, L138A, and A705I even showing improved efflux of NPN, compared to the WT (Figure 3B). All of these mutants were like the WT pump in efflux of EtBr (Figure S7B).

**Figure 3:**
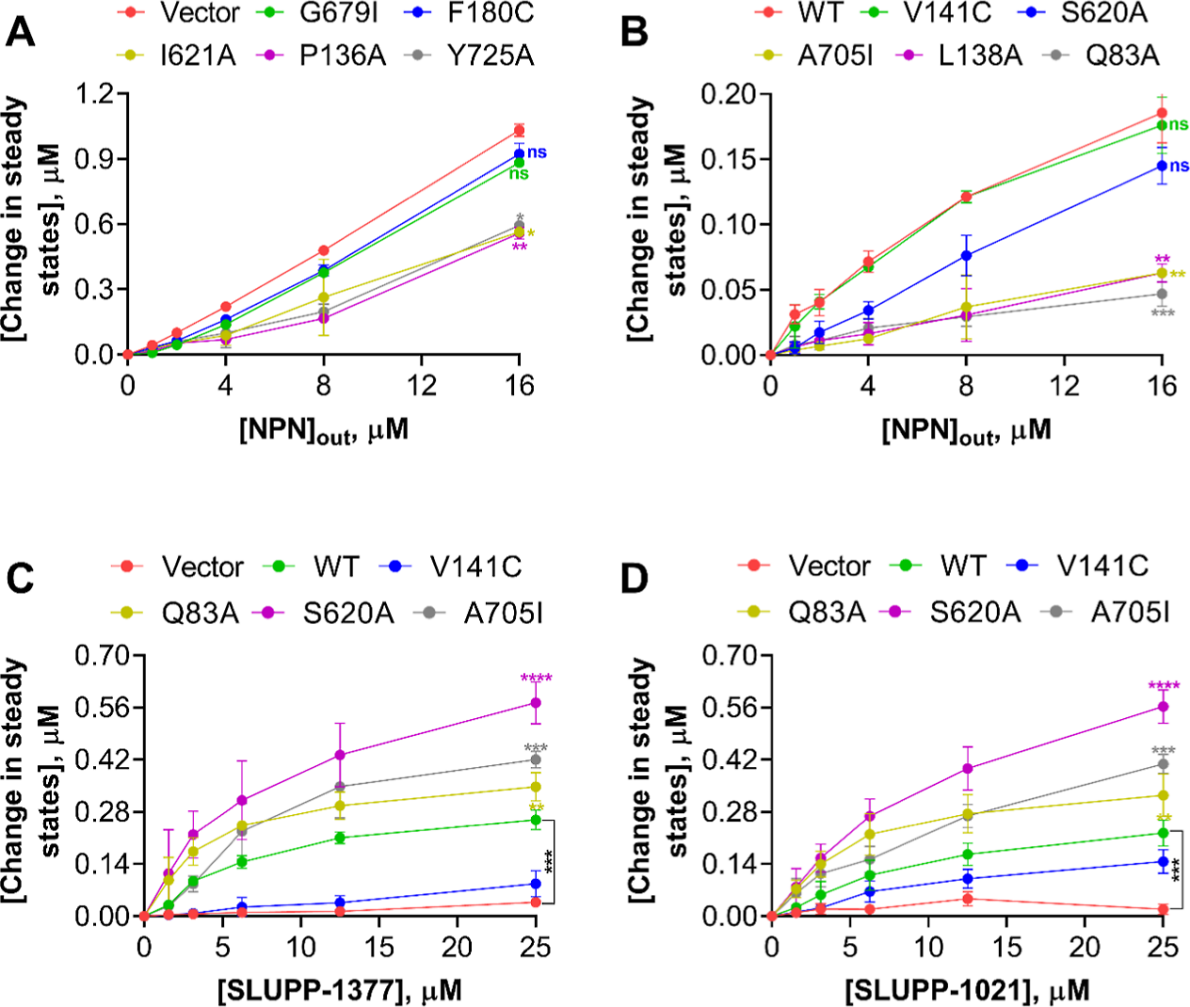
(A,B) Steady-state NPN accumulation levels in *A. baumannii* Δ3-pore cells carrying an empty vector, WT (AdeFGH) pump, and indicated AdeG single amino acid substituted variants, calculated from the kinetic curves shown in Figure S5. NPN was used at the final external concentration ranging from 0 μM-16 μM. Panel A represents AdeG variants with impaired efflux ability, like the empty vector, and panel B represents similar NPN accumulation levels as WT. (C,D) Steady-state NPN (8 μM external concentration) accumulation levels calculated from the kinetic curves shown in Figure S6 (SLUPP-1377) and Figure S7 (SLUPP-1021). The EPIs were used at a concentration ranging from 0 μM-25 μM. Each data point represents the average of two biological replicates with two technical repeats ±SD. Data was analyzed by a *t*-test. Values were considered statistically significant at *P < 0.05 and highly significant at **P < 0.01, ***P < 0.001, and ****P < 0.0001. ^#^ns, nonsignificant.

### DAQs Engage Similar Contacts in the Distal Binding Pockets of AdeG and AdeJ but Not in the Proximal Binding Pockets

To determine how mutations in AdeG affect the activity of **SLUPP-1377** and **SLUPP-1021**, we first determined MPC_4_ values of the inhibitors in the presence of antibiotics on the substrates of AdeG and then analyzed the kinetics of intracellular accumulation of NPN in cells producing AdeG variants. Both inhibitors were the most effective in combination with NOR and NAL, with the MPC_4_ values at 8- to 16-fold lower than MICs of the inhibitors in cells producing the wild-type AdeFGH but not in the efflux-deficient AbΔ3-pore cells carrying an empty vector (Table 4). For the cells carrying the nonfunctional AdeG F180C (in the DBP) and G679I (F-loop) variants, the MPC_4_ values of the inhibitors were equal to or within 2-fold of the MIC values of the inhibitors in the corresponding cells and equal to those of the efflux-deficient control.

**Table 4:** MPC_4_ of Naphthyl-Substituted DAQs with Substrate Antibiotics in the Efflux-Deficient *A. baumannii* ATCC17978 Strain Carrying either an Empty Vector (Vector) or Producing the WT and the Indicated AdeG Variants

			MPC_4_ (μM)					
	MICs (μM)		SLUPP-1377			SLUPP-1021		
strains	SLUPP-1377	SLUPP-1021	CHL	NOR	NAL	CHL	NOR	NAL
vector	6.25	25	6.25	6.25	6.25	25	25	25
WT	25	50	6.25	3.125	3.125	12.5	3.125	6.25
G679I	12.5	25	12.5	6.25	6.25	25	12.5	12.5
Q83A	50	100	6.25	1.56	3.125	12.5	3.125	6.25
A705I	25	50	6.25	1.56	1.56	12.5	3.125	6.25
Y725A	25	50	12.5	6.25	6.25	25	12.5	25
S620A	25	50	6.25	**0.78** ^**a**^	1.56	12.5	1.56	3.25
I621A	25	50	12.5	6.25	12.5	25	12.5	25
P136A	12.5	25	6.25	6.25	6.25	12.5	12.5	12.5
L138A	50	100	6.25	3.125	6.25	12.5	6.25	12.5
V141C	25	50	**25**	**25**	**25**	**50**	**25**	**50**
F180C	12.5	25	12.5	6.25	6.25	25	12.5	12.5

In contrast, **SLUPP-1377** potentiated the activity of NOR in AdeG S620A (G-loop), A705I, and Q83A (both in PBP) with MPC_4_ values 2–4-fold lower than the **SLUPP-1377** MPC_4_ value in AbΔ3­(pAdeFGH) cells producing the WT AdeG, suggesting that these three mutant pumps are hypersensitive to the action of the inhibitor (Table 4). The effect of S620A, A705I, and Q83A substitutions on MPC_4_ values of **SLUPP-1021** in combination with NOR and other antibiotics was less noticeable, within a 2-fold difference (Table 4). Likewise, these three AdeG variants were more sensitive to the inhibitory action of **SLUPP-1377** and **SLUPP-1021** in the NPN and EtBr accumulation assays (Figures 3C,D and S8–S10).

The AdeG V141C (DBP) variant was distinct from other variants because both **SLUPP-1377** and **SLUPP-1021** failed to potentiate the activities of tested antibiotics in cells overproducing this variant (Table 4). This result suggested that the V141C-substitution leads to resistance against the inhibitors. The V141C resistance to **SLUPP-1377** is also seen in the checkerboard assay (FICI = 1.25 suggests no interaction or indifference), in which both the concentrations of NOR and the inhibitor vary (Figure 4) and in the NPN accumulation assay, in which increasing concentrations of **SLUPP-1377** fail to increase the intracellular accumulation of the substrate (Figure 3C). The effect of V141C-substitution on the inhibitory activity of **SLUPP-1021** in the NPN assay was also notable but not as strong as in the combination with **SLUPP-1377** (Figure 3D), and this substitution had no effect on the activity of **SLUPP-1377** against the efflux of EtBr (Figure S10). No differences were found in the MPC_4_ values of other AdeG mutants versus the WT pump.

**Figure 4:**
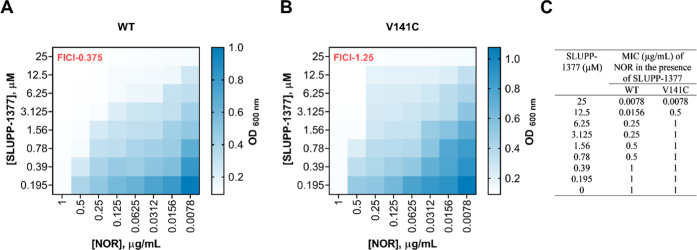
(A,B) Synergistic activity between SLUPP-1377 and substrate NOR by the checkerboard assay against *A. baumannii* Δ3-pore cells overproducing AdeFGH (WT) and AdeG mutant V141C. Dark-colored regions represent higher cell density. Data represents the OD_600 nm_ of cells in the 96-well microtiter plates after 20–24 h of incubation with shaking. FICI values represent synergy (0.375) between SLUPP-1377 and NOR for WT and no interaction or indifference (1.25) for AdeG variant V141C. (C) MICs of NOR in the presence of SLUPP-1377 (0 μM-25 μM) against *A. baumannii* Δ3-pore cells overproducing AdeFGH (WT) and AdeG mutant V141C in the checkerboard assay.

Interestingly, we previously found that the analogous mutations in AdeJ had a different effect on the activity of **SLUPP-1021** when used in combination with AdeJ substrates such as novobiocin, erythromycin, and tetracycline (see compound **2** in ref 22). In all of these combinations, only AdeJ F178C (F180C in AdeG) was hypersensitive to the activity of **SLUPP-1021**, whereas no other differences from the WT AdeJ were found for other variants. We, therefore, analyzed the sensitivity of AdeJ variants to the action of **SLUPP-1021** and **SLUPP-1377** in combinations with NOR and CHL, which are also recognized by AdeIJK as substrates. We found that only AdeJ F178C (DBP) was hypersensitive to the action of these inhibitors in combinations with NOR and CHL, as seen with other substrates of AdeJ, but not the variants with substitutions in PBP or loops (Table S2). Surprisingly, the AdeJ V139C (DBP) variant was similar to its AdeG V140C analogue and became more resistant to all combinations (Table S2). The effect of the V139C-substitution in AdeJ on the inhibitory activity of **SLUPP-1377** in the NPN assay was also obvious (Figure S11). Thus, these two inhibitors engage similar interactions in the DBP of both AdeG and AdeJ pumps but differ in interactions with the PBP and the interface regions.

## Discussion

*A. baumannii* MDR strains overproducing RND efflux pumps have become one of the most problematic pathogens in clinics.^26–28^ Among the three major RND pumps, the role of AdeFGH in antibiotic resistance and physiology of *A. baumannii* remains unclear.^29^ Even significant overproduction of AdeFGH due to regulatory mutations or by induction of the plasmid-borne gene expression (Figures 1A and 2B) results only in a modest 4–8-fold increase in MICs of substrate antibiotics (Tables 1, 3, and S5), and the range of affected antibiotics is relatively narrow.^20^ These changes in antibiotic susceptibilities fade in comparison to the changes due to overproduction of AdeIJK or AdeABC pumps.^19^ Finding inhibitors specific for the AdeFGH pump could facilitate the understanding of the role of AdeFGH in *A. baumannii* antibiotic resistance and physiology and the mechanistic differences between AdeFGH and the related efflux pumps.

Compounds **SLUPP-1377** and **SLUPP-1021** from the DAQ series were previously found to enhance the activity of novobiocin and other antibiotics in AdeIJK overexpressing and WT *A. baumannii* strains without permeabilizing bacterial membranes.^18,22^ Here, we characterized the activity of these compounds against AdeFGH and identified similarities and differences between AdeG and AdeJ in how specific residues within their ligand translocation path interact with substrates and the inhibitors. We found that both compounds are substrates of AdeG and inhibit the activity of the pump. **SLUPP-1377** is more effective than **SLUPP-1021** against both the plasmid-borne and mutationally overproduced AdeG in various genetic backgrounds (Figure 1) and in combinations with antibiotics CHL, NOR, and NAL, which are the substrates of both AdeG and AdeJ pumps. Our results further show that these inhibitors do not potentiate the antibacterial activities of antibiotics in cells lacking AdeFGH and increase the intracellular accumulation levels of substrates of AdeFGH, and their inhibitory activities respond to point mutations in AdeG. These results strongly suggest that the activities of these inhibitors are dependent on the activity of AdeFGH and establish **SLU-1377** and **SLUPP-1021** compounds as broad-spectrum inhibitors of RND pumps.

The overall structures of AdeG and AdeJ are remarkably similar to each other and other characterized RND pumps (Figure 5).^9^ The four conserved regions, the F-loop, PBP, G-loop, and DBP, contribute to substrate specificities and create gated tunnels in RND pumps that facilitate the translocation of various ligands by the transporters. Of the ten constructed single-substitution AdeG mutants, AdeG G679I (F-loop) and F180C (DBP) both appeared to be nonfunctional mutants since they exhibited efflux activity levels that were close to the negative control strain AbΔ3-Pore. The G679 residue in the F-loop, which separates the ligand entrance site from the PBP, is not conserved between the pumps, and the substitutions in these positions in AdeG and AdeJ have the substrate-specific and opposing effects (Figure 5, Table S6). Despite the different properties of the acidic glutamate residues in AdeB and AdeJ and the glycine in AdeG, the negative effects of substitutions in this position are observed in all three Ade pumps (Table S7). However, direct comparison of substrate specificities showed that AdeJ and AdeG are more efficient in the efflux of fluoroquinolones and CHL than AdeB.^20^ Together, these findings suggest that the effect of substitutions on the substrate specificity is indirect and might involve changes in the conformation and/or flexibility of the F-loop. Interestingly, E675A substitution in AdeJ led to resistance against phenyl-substituted DAQs but not against the naphthyl-substituted ones such as **SLUPP-1021**.^18^ Thus, both the state of the F-loop as well as physicochemical features of compounds define the outcomes of specific single amino acid substitutions in this region.

**Figure 5:**
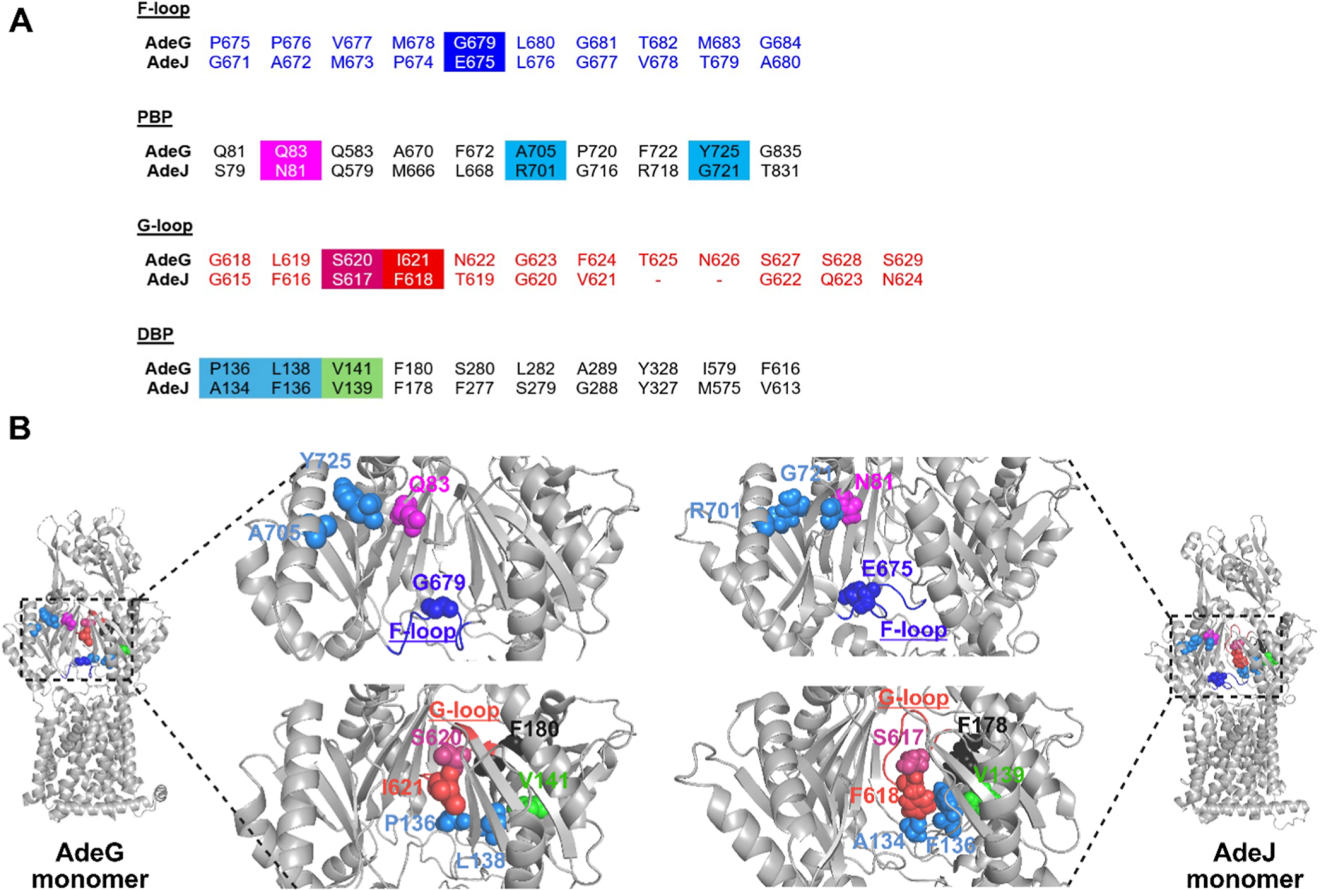
Comparison of the multidrug binding sites between AdeG and AdeJ. (A) Pairwise sequence alignment of the substrate and/or inhibitor binding sites of AdeG and AdeJ. For both AdeG (this study) and AdeJ^22^ pumps, the highlighted amino acids in the F-loop, PBP, G-loop, and DBP have been selected for mutagenesis. The homologous amino acid sites in both pumps are represented with the same color. (B) Structure of AdeG (PDB ID: 8YR0)^9^ and AdeJ (PDB ID: 7M4Q)^23^ binding monomers with zoomed-in views of PBP with the F-loop and DBP with the G-loop. The amino acid residues in the substrate and/or inhibitor translocation path are shown as spheres in different colors. The comparison between homologous sites in AdeG and AdeJ has been made based on the inhibitory activities of SLUPP-1377 and SLUPP-1021 described in Table S6. The amino acids in AdeG and AdeJ with marine blue color represent similar MIC/MPC_4_ ratios (AdeG vs AdeJ: A705 vs R701, Y725 vs G721, P136 vs A134, and L138 vs F136). Other amino acids with different colors represent changes in MIC/MPC_4_ ratios. AdeG vs AdeJ: G679 vs E675 (blue), Q83 vs N81 (magenta), S620 vs S617 (warm pink), I621 vs F618 (red), and V141 vs V139 (green).

Interactions with the PBP have been implicated in the recognition of both substrates and inhibitors, and these interactions define whether the ligands are translocated further through the G-loop or dissociate from the pump. The Y725A substitution in the PBP of AdeG but not Q83A and A705I reduced the activity of the pump (Table 3) but did not affect the activities of inhibitors (Table 4). Although the analogous substitutions in AdeJ had no effect on the efflux of substrates and activities of the naphthyl-substituted **SLUPP-1377** and **SLUPP-1021**, like E675A, the R701A substitution in AdeJ provided resistance against the phenyl-substituted DAQ series.^22^

The I621A in the G-loop of AdeG reduced efflux of substrates and did not affect the activity of DAQs, whereas the adjacent S620A increased sensitivity to inhibitors, particularly in the combination with NOR and in the efflux of NPN (Tables 3 and 4 and Figure 3A,C). Both substitutions did not change dramatically the size or properties of the AdeG residues. However, in the position analogous to I621 of AdeG, AdeJ contains a bulky phenylalanine F618 and its replacement with alanine also had only minor effects on efflux activities, pointing to significant flexibility in the properties of amino acid residues forming the G-loop.

Once past the G-loop, the ligands enter DBP and can interact with its hydrophobic trap. The aromatic group of F178 was found to form stacking interactions with eravacycline and fluorocycline ligands in the DBP of AdeJ,^30^ EtBr in AdeB,^31^ and several ligands in AcrB.^32,33^ It is also a part of the hydrophobic trap, the site within the DBP where inhibitors were reported to bind in the homologous *E. coli* AcrB^34^ and *P. aeruginosa* MexB.^35^ Although both AdeG F180C and AdeJ F178C variants were partially defective in efflux activities, F178C-substitution in AdeB resulted in the hyposusceptibility against several antibiotics, indicating improved efflux activity (Table S6).^36^ The effect of these substitutions on the sensitivity to inhibitors was also specific to the pump. The F178C-substitution made AdeJ hypersensitive to the action of DAQs, whereas the AdeG F180C variant was nonsusceptible to DAQs (Figure 5, Table S6). Interestingly, the L138A substitution in AdeG and its corresponding F136A in AdeJ, both located in the DBP, improved the efflux of select substrates but did not modulate the inhibitory activities of DAQs. On the other hand, the V141C-substitution in DBP of AdeG and V139C of AdeJ provided resistance against DAQs in both bacterial growth-dependent and -independent assays (Figures 3A,B and 5; Table S6 and Figure S11). This finding suggests that DAQs compete with NOR, CHL, and NPN, but not other substrates of AdeJ, for interaction with V139 in the DBP, which could lead to substrate-specific resistance to DAQs when V139 is substituted.

Taken together, our results suggest that substrates and inhibitors share similar contacts in the DBPs of AdeG and AdeJ pumps, which are likely to enable the broad-spectrum activity of DAQs. However, the two pumps differ in how they interact with the ligands during entrance and recognition in the PBP. The impact of specific amino acid substitutions in AdeG and AdeJ is context-dependent on both the structures of ligands and the composition of the entrance and the PBPs and likely to be modulated through the long-range interactions of substrates and inhibitors within the translocation paths of the pumps. Further studies are needed to establish molecular details of efflux pump interactions with inhibitors and to develop narrow-spectrum pump-specific inhibitors without significant cytotoxicity.

## Experimental Procedures

### Antimicrobial Susceptibility Testing

The strains and plasmids used in this study are listed in Table 5. All bacterial strains were grown in Luria–Bertani (LB) broth at 37 °C with aeration. The susceptibilities of *A. baumannii* strains to different antibiotics and EPIs were determined by a 2-fold broth microdilution method as described previously.^18^ The lowest concentration of a drug that inhibits visible growth is defined as the MIC. In agreement with previous studies,^37^ we found that MICs of AZM depended on the growth medium, with the values in LB (2 μg/mL in ATCC17978) being 16–32-fold lower than in cation-adjusted Mueller–Hilton Broth (32–64 μg/mL in the same strain).

**Table 5:** Strains and Plasmids Used in This Study

strains	relevant genotype	source
ATCC17978	drug-susceptible A. baumannii	ATCC
JWW30	A. baumannii ATCC17978 (Str^r^)	^40^
IL119 (AbΔ3)	JWW30 Δ*adeIJK*Δ*adeAB*Δ*adeFGH*	^40^
IL 122(AbΔ3) (vector)	IL119 *att*Tn7::miniTn7T-Tmp^r^-*araC*-P_*BAD*_ -MCS carrying pTJ1	^36^
IL139 (AbΔ3-pore)	JWW30 Δ*adeAB*Δ*adeFGH*Δ*adeIJK* *att*Tn7::mini- Tn7T-Kan^r^-*araC*-P_*BAD*_-FhuA	^20^
IL161(AbΔ3-pore) (vector)	IL139 *att*Tn7::miniTn7T- Kan^r^-*araC*-P_*BAD*_-FhuA carrying pTJ1	^36^
IL141(AbΔ3 pAdeFGH) (WT)	L119 *att*Tn7::mini-Tn7T-Tmp^r^-*araC*-P_*BAD*_-*adeFGH* carrying pTJ1-*adeFGH*	^20^
IL147(AbΔ3 pAdeFGH-pore) (WT)	IL139 *att*Tn7::mini-Tn7T-Kan^r^-*araC*-P_*BAD*_-FhuA carrying pTJ1-*adeFGH*	^20^
IL14(AbΔ3 pAdeIJK-pore) (WT)	IL139 *att*Tn7::mini-Tn7T-Kan^r^-*araC*-P_*BAD*_-FhuA carrying pTJ1-*adeIJK*	^20^
AbΔ3 (pAdeIJ*K)-pore	IL139 *att*Tn7::mini-Tn7T-Kan^r^-*araC*-P_*BAD*_-FhuA carrying pTJ1-adeIJ*K (*AdeJ single amino acid substituents- E675A, N81A, R701A, G721I, F618A, A134I, F136A, V139C, F178C)	^22^
AYE	MDR clinical isolate	^42^
AYE Δ2	AYE Δ*adeB*Δ*adeIJK*	^19^
AYE Δ2 AdeL^T319 K^	AYE Δ2 spontaneous Chl^r^ variant with a substitution mutation in AdeL (threonine to lysine at residue 319)	this study
Ab5075	MDR clinical isolate	^43^
Ab5075 Δ2	Ab5075 *adeB*::T26Δ*adeIJK*	^19^
Ab5075 Δ2 AdeL^V139G^	Ab5075 Δ2 spontaneous Chl^r^ variant with a substitution mutation in AdeL (valine to glycine at residue 139)	this study
Ab5075 Δ2 AdeL^IS5^	Ab5075 Δ2 spontaneous Chl^r^ variant with an inactivation of AdeL due to insertion of IS5-like transposons	this study
Escherichia coli C43 (DE3)	F—*ompT* *hsdSB* (rB- mB-) *gal dcm* (DE3) Δ*acrB*	^44^
pTNS3	Amp^r^; helper plasmid expressing *tnsABCD* (Tn7 transposase) from *P1* and *P*_*lac*_	^45^
pET-21 a (+)	Amp^r^; T7_lac_, T7 (N-term), his (C-term)	novagen
pIL 153 (pET-21 a (+)-*adeG*)	Amp^r^; *adeG* cloned between NdeI and XhoI restriction sites	this study
pTJ1	pUC18T-mini-Tn7T-Tmp-*araC*-P_*BAD*_-MCS, Amp^r^, Tmp^r^	^46^
pIL 130 (pTJ1-*adeFGH*)	pUC18T-mini-Tn7T-Tmp-*araC*-P_*BAD*_ *-adeFGH*, Amp^r^, Tmp^r^	^20^
pTJ1-AdeFG^#^H	pUC18T-mini-Tn7T-Tmp-*araC*-P_*BAD*_ *-adeFG* ^*#*^ *H*, Amp^r^, Tmp^r^ (^#^AdeG single amino acid substituents—G679I, Q83A, A705I, Y725A, S620A, I621A, P136A, L138A, V141C, F180C)	this study

The synergistic interactions between antibiotics and EPIs were assessed by a checkerboard titration assay according to a protocol published elsewhere.^18,22^ The MPC_4_ of an EPI was calculated, defined as a concentration of the test compound that decreases the MIC of an antibiotic in combination by four-fold. To assess synergy, the fractional inhibitory concentration index (FICI) was calculated.

### Development of Spontaneous CHL-Resistant Mutants

To select AYE Δ2 and Ab5075 Δ2 CHL^r^ clones, 50 μL of concentrated exponential phase cultures (OD_600 nm_ = 10) was plated onto LB agar containing CHL at a concentration range of 1 × MIC – 8 × MIC. Resistant colonies appeared within 24 h of incubation at 37 °C. For the initial confirmation, several colonies were inoculated into the LB medium without selection pressure and the MIC was determined. The colonies with at least a 4-fold increase in MICs with respect to their parental strains were further subjected to colony PCR with gene (*adeG* and *adeL*)-specific primers and sequencing for final validation.

### Time-Kill Kinetics

The overnight-grown Ab5075 Δ2 AdeL^IS5^ cells were diluted to 10^6^ CFU/mL in fresh LB medium and treated with either NOR (1 × MIC) or SLUPP-1377 (MPC_4_) alone or in combination. The untreated bacterial suspension served as a control. The different treatment sets were incubated at 37 °C for 24 h with shaking. A 100 μL portion of the culture sample was withdrawn at different time points, appropriately diluted in 1× phosphate-buffered saline (PBS, pH 7.4), and spotted (10 μL) on LB agar. After 24 h, the colonies were counted and log_10_ CFU/mL was plotted against time.

### Fluorescent Probe Uptake

The cells for the uptake of fluorescent probes were prepared as described previously.^18,22^ NPN and EtBr (0 μM-16 μM final concentration) were diluted in a black 96-well nonbinding microplate (Greiner Bio-One) containing HMG buffer in a total 100 μL volume. Then, 100 μL of cells was injected using a TECAN Spark multimode microplate reader. For EPIs (0 μM–25 μM final concentration), NPN and EtBr were used at a constant concentration of 8 and 4 μM, respectively. The fluorescence intensity was immediately monitored before and after addition of cells at λ_ex_-350 nm and λ_em_-405 nm for NPN and λ_ex_-480 nm and λ_em_-610 nm for EtBr. The relative fluorescence intensities were plotted against time, the data was exported to MATLAB (MathWorks) to be fitted to a simple exponential equation, and the steady-state concentrations were calculated and plotted against the external concentration of NPN, EtBr, and EPIs.^38^

### Measurement of NOR Accumulation

The accumulation of norfloxacin was determined as previously described,^39^ with the following modifications. Bacterial cultures were grown to an OD_600 nm_ = 0.5 in the LB medium. The concentrated exponential phase (OD_600 nm_ = 10) cultures were treated with NOR (16 μg/mL) on ice for 5 min. For the EPI treatment, samples were treated with **SLUPP-1377** (25 μM) for 15 min at room temperature in the LB medium prior to NOR treatment. The samples were then centrifuged and washed twice with ice-cold phosphate buffer (pH 7.0). The samples were then resuspended in 1 mL of 100 mM glycine-HCl buffer (pH 3.0) and incubated for 2 h at room temperature. Then, samples were centrifuged, and the fluorescence (λ_ex_-281 nm and λ_em_-440 nm) of the supernatant was recorded.^39^

### Site-Directed Mutagenesis

All substitutions in the *adeG* gene were constructed using QuikChange XL site-directed mutagenesis kit (Agilent) and Q5 site-directed mutagenesis kit (New England Biolabs) according to the manufacturer’s instructions using pIL 130 (pTJ1-*adeFGH*) as a template. Introduced substitutions and the lack of undesired mutations were verified by whole plasmid sequencing (Plasmidsaurus). These plasmids were inserted into AbΔ3 and AbΔ3-pore strains as described previously.^20,40^

### Production of Polyclonal Anti-AdeG Antibodies

The *adeG* gene was amplified by PCR using the genomic DNA of the *A. baumannii* WT (JWW30) strain as a template and cloned into pET-21 a (+) with NdeI and XhoI as restriction sites to generate pET-21 a (+)-*adeG* expressing the efflux transporter under the control of the isopropyl β-d-1-thiogalactopyranoside (IPTG)-inducible promoter. To purify AdeG, *E. coli* C43-AdeG cells were cultured in the LB medium containing ampicillin (100 μg/mL) and induced with 0.1 mM IPTG for an additional 5 h at an OD_600 nm_ of ∼0.3. Protein purification was done as described previously.^41^ Purified AdeG was visualized on 8% sodium dodecyl sulfate-polyacrylamide gel electrophoresis (SDS-PAGE), stained with Coomassie Brilliant Blue, and cut from the gel. The gel lane with purified AdeG (∼3.6 mg) was suspended in 1× PBS and sent to Thermo Fisher Scientific for immunization and polyclonal anti-AdeG antibody production in rabbits.

### Protein Expression Analyses

Overnight, *A. baumannii* cells were inoculated from frozen cell stocks. The cells (2% v/v) were subcultured in 20 mL of fresh LB medium and incubated at 37 °C to an OD_600 nm_ of 0.25–0.3. The cells were induced with 1% l-arabinose (when specified) and further incubated for 3 h. The OD_600 nm_ values of all the cells were adjusted to equal cell density. The cells were collected by centrifugation at 3175*g* for 20 min at room temperature. The pellet was resuspended in 1 mL of buffer (10 mM Tris, pH 8; 150 mM NaCl; 1 mM MgCl_2_; 50 μg/mL DNase I; 50 μg/mL Lysozyme; 1 mM PMSF), incubated for 30 min on ice, and subjected to sonication for lysis. The cell lysates were centrifuged at 2348*g* for 5 min at 4 °C to remove the cell debris. The equal volume of cell supernatants was loaded and separated onto 10% SDS-PAGE, transferred to a PVDF (Immobilon-FL) membrane for immunoblotting with primary anti-AdeG polyclonal antibodies (Thermo Fisher Scientific) and a secondary alkaline phosphatases-conjugated antirabbit antibody (Sigma). 5-Bromo-4-chloro-3-indolyl phosphate and nitro blue tetrazolium were used to visualize the protein bands. The image acquisition and relative quantification of protein expression were done using Quantity One 1-D analysis software.

### Statistical Analyses

Statistical analyses were performed using GraphPad Prism (version 8.0.2) software. The data were plotted as mean ± SD. A two-tailed *t*-test was used to compare the two groups. Values were considered statistically significant at *P < 0.05 and highly significant at **P < 0.01, ***P < 0.001, and ****P < 0.0001.

## Supplementary Material



## Notes

Kinetics and calculated steady-state levels of intracellular uptake of NPN in *A. baumannii* MDR and Δ3 cells and their derivatives in the absence and presence of **SLU-1377** and **SLU-1021**; immunoblotting analysis of cell lysates from *A. baumannii* Δ3-pore cells carrying an empty vector, WT pump, and AdeG single amino acid-substituted variants; kinetics and calculated steady-state concentrations of intracellular uptake of NPN in *A. baumannii* Δ3 cells carrying AdeG mutant variants in the absence and presence of **SLU-1377** and **SLU-1021**; quantitation of relative intensities in immunoblotting analysis of AdeG expression in various strains; MICs of substrate antibiotics and MPC_4_ of EPIs in strains producing the WT (AdeIJK) efflux pump and AdeJ mutant variants with the native and hyperporinated outer membrane; FICI of naphthyl-substituted DAQs with substrate antibiotics; and comparisons of the impact of single amino acid substitutions in the multidrug binding sites of AdeG, AdeB, and AdeJ on the inhibition abilities of EPIs and transporter’s activities (PDF)
