# Supplementary material for: Broad-Spectrum Naphthyl-Substituted Diaminoquinolines Inhibiting the AdeG Efflux Pump of Acinetobacter baumannii
Source: ACS Infect Dis. 2026 Jan 5;12(2):588–99. doi: 10.1021/acsinfecdis.5c00722 (PMC12910579; doi:10.1021/acsinfecdis.5c00722)
Supplement: Supplementary file 1 [file id5c00722_si_001.pdf]

Supporting Information

Broad spectrum naphthyl-substituted diaminoquinolines inhibiting AdeG efflux pump of *Acinetobacter baumannii*

Rushikesh Tambat[1], Aysegul Saral Sariyer[2], Emrah Sariyer[2,3], Marcela Olvera[1], Mithila Farjana,[1] Napoleon D'Cunha[4], John K. Walker,[4,5] Helen I. Zgurskaya[1,*]

[1]University of Oklahoma, Department of Chemistry and Biochemistry, Norman, OK 73019, USA

[2]Artvin Coruh University, Department of Nutrition and Dietetics, Faculty of Health Sciences, 08000, Artvin, Turkey

[3]Artvin Coruh University, Vocational School of Health Services, Medical Laboratory Techniques, 08000, Artvin, Turkey

[4]Saint Louis University School of Medicine, St. Louis, MO 63110, USA

[5]Saint Louis University, Department of Chemistry, St. Louis, MO 63110, USA

[*]Corresponding author: elenaz@ou.edu

Keywords: *Acinetobacter baumannii*, multidrug efflux pumps, efflux pump inhibitors

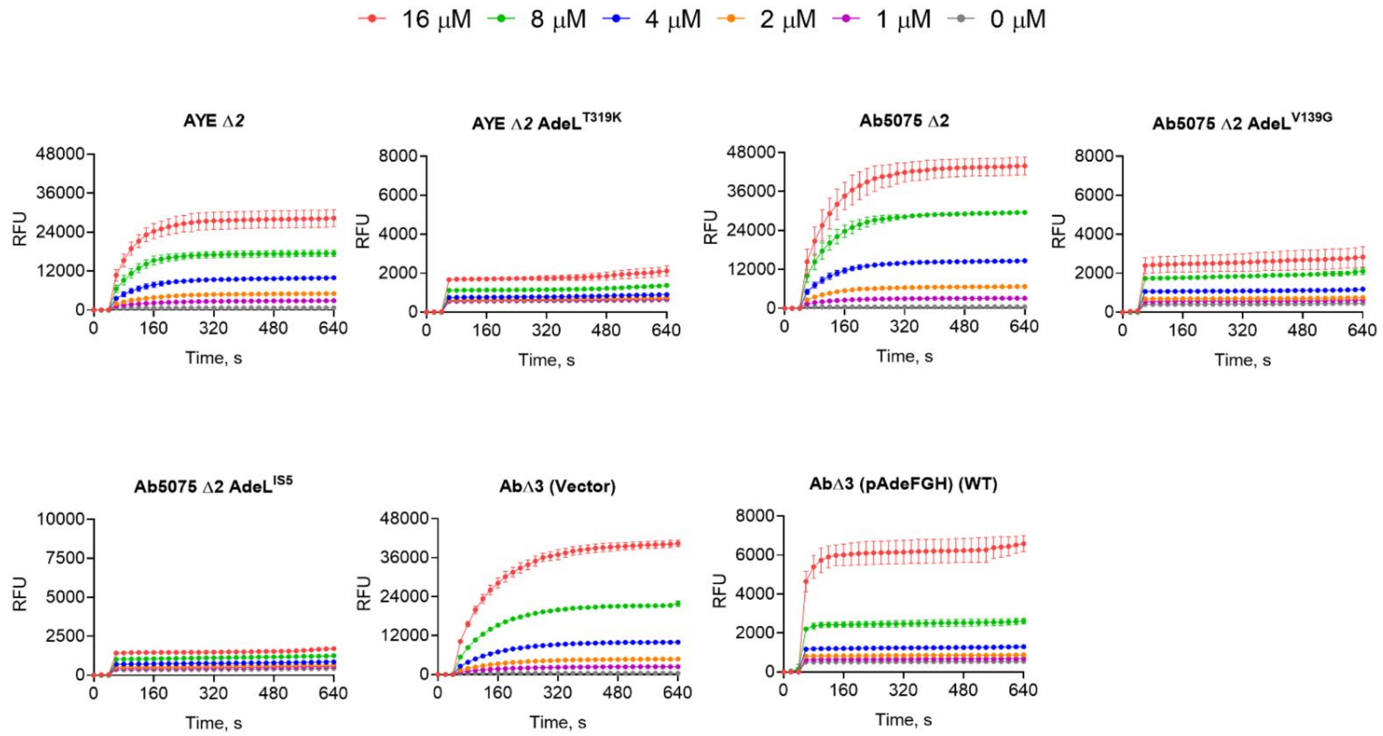

Figure S1. Intracellular uptake of *N*-Phenyl-1-naphthylamine (NPN) in *A. baumannii* multidrug resistant (MDR; AYE and Ab5075) and Δ3 cells with its indicated variants. Data represent real-time changes in NPN (0 μM-16 μM final external concentration) fluorescence. Relative fluorescence units (RFUs) were calculated by normalizing fluorescence intensity after subtracting background fluorescence without cells and plotted against time.Each data point represent the average of two biological replictates with two technical repeats ± standard deviation (SD).

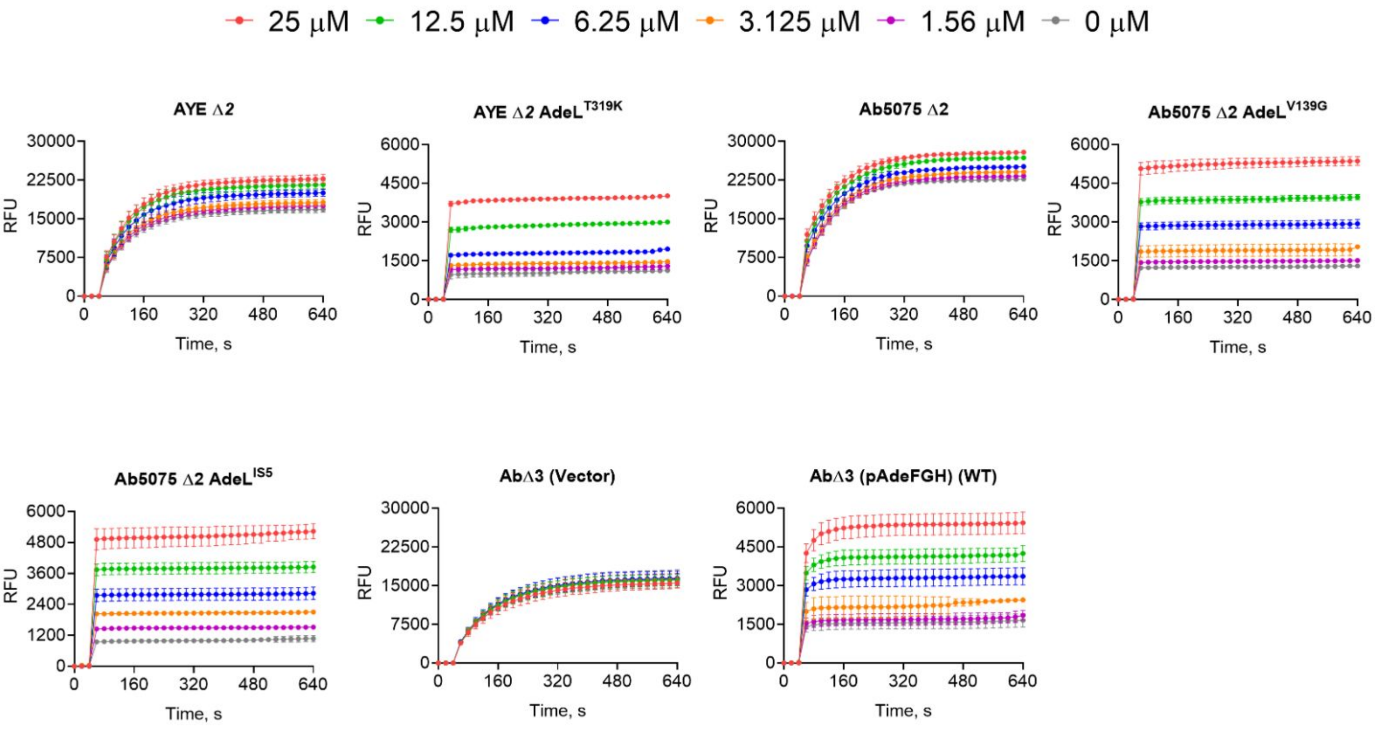

Figure S2. Kinetic curves of NPN uptake (8 μM final external concentration) in *A. baumannii* MDR cells and their indicated variants. All cells were treated with SLUPP-1377 (0 μM-25 μM final external concentration) during kinetics. Each data point represent the average of two biological replictates with two technical repeats ± SD.

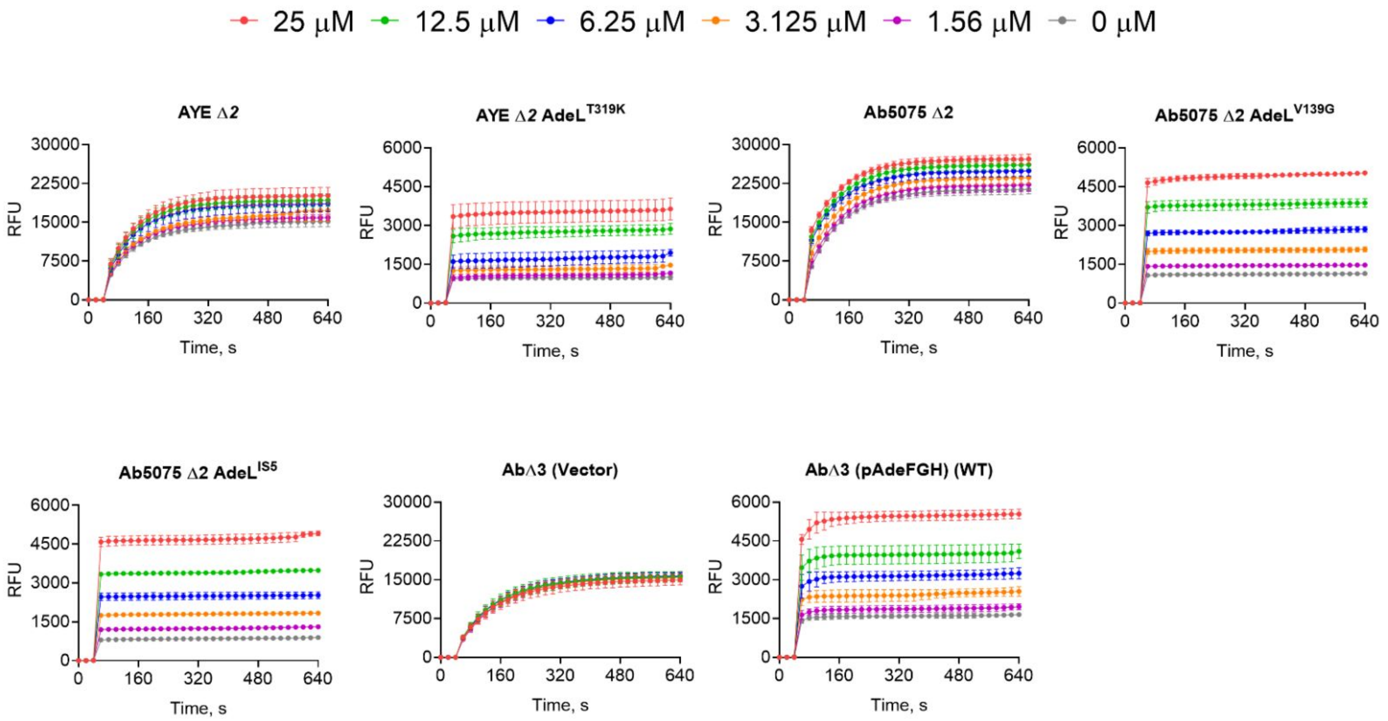

Figure S3. Kinetic curves of NPN uptake (8 μM final external concentration) in *A. baumannii* MDR cells and their indicated variants. All cells were treated with SLUPP-1021 (0 μM-25 μM final external concentration) during kinetics. Each data point represent the average of two biological replictates with two technical repeats ± SD.

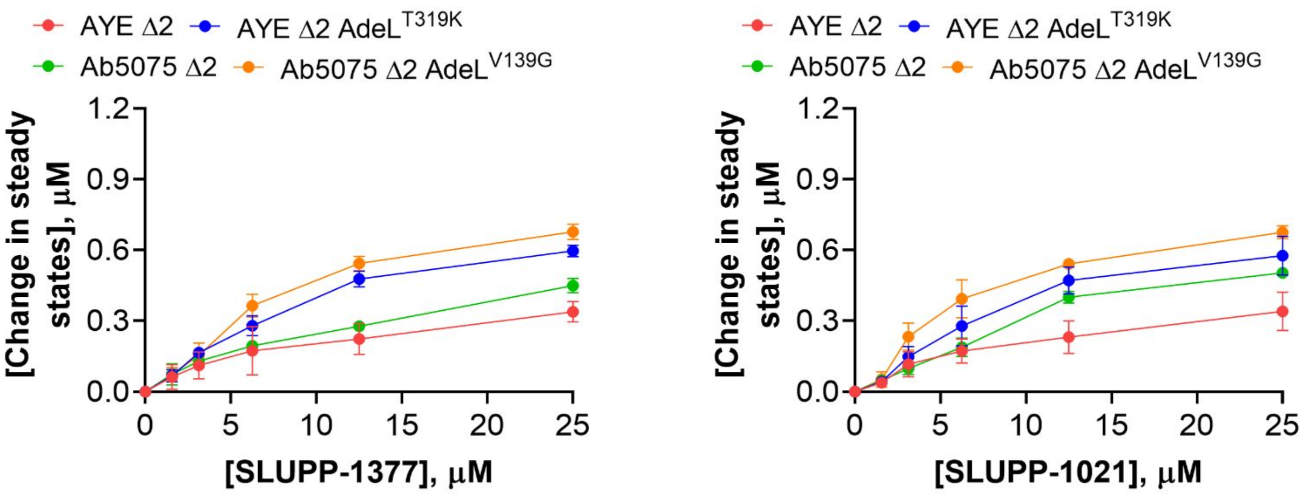

Figure S4. Steady-state NPN (8 μM external concentration) accumulation levels calculated from the kinetic curves shown in Figure S2 (SLUPP-1377) and Figure S3 (SLUPP-1021). The EPIs were used at the concentration ranging from 0 μM-25 μM. Each data point represents the average of two biological replicates with two technical repeats ± standard deviation (SD).

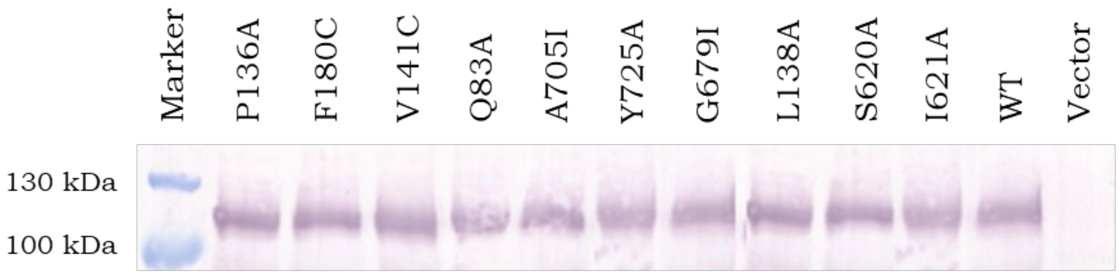

Figure S5. Immunoblotting analysis of cell lysates from *A. baumannii* Δ3-pore cells carrying an empty vector, WT (AdeFGH) pump, and indicated AdeG single amino acid substituted variants. AdeG (~114.1 kDa) variants were visualized with an anti-AdeG polyclonal antibody.

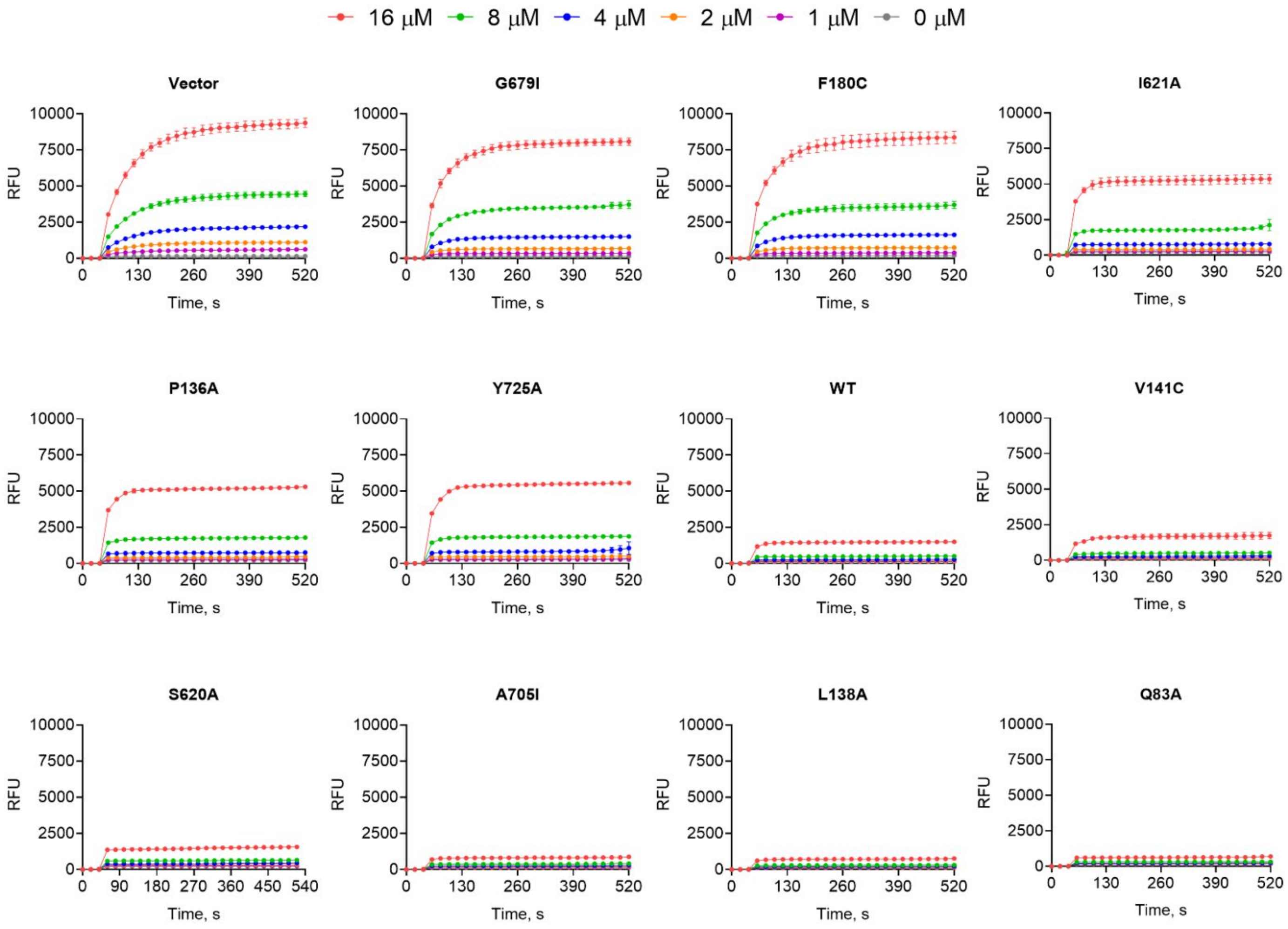

Figure S6. Intracellular uptake of NPN (0 μM-16 μM final external concentration) in *A. baumannii* Δ3-pore cells carrying an empty vector, WT (AdeFGH) pump, and indicated AdeG single amino acid substituted variants. Each data point represent the average of two biological replictates with two technical repeats ± SD.

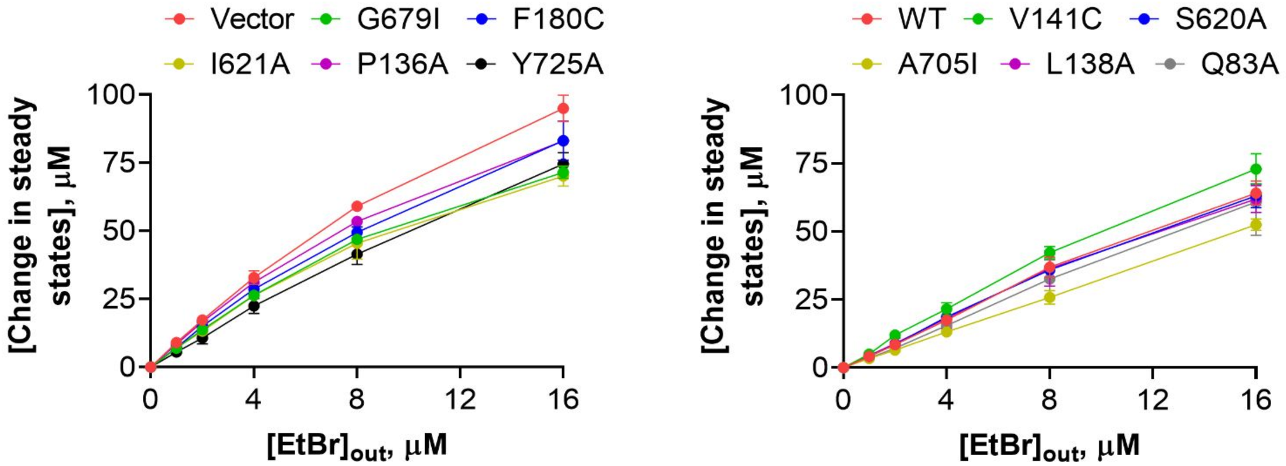

Figure S7. Steady-state ethidium bromide (EtBr) (0 μM-16 μM final external concentration) accumulation levels in *A. baumannii* Δ3-pore cells carrying an empty vector, WT (AdeFGH) pump, and indicated AdeG single amino acid substituted variants. Each data point represent the average of two biological replictates with two technical repeats ± SD.

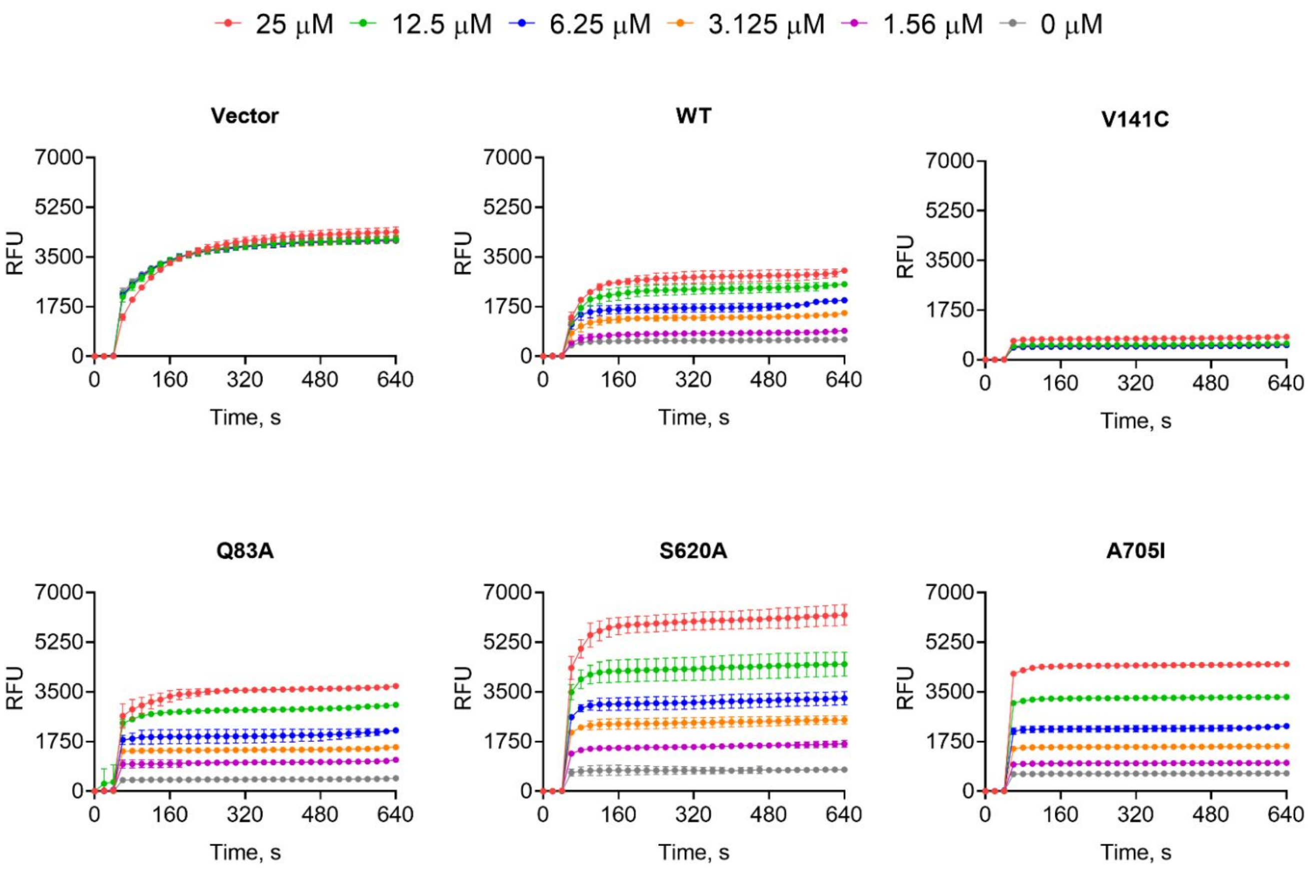

Figure S8. Kinetic curves of NPN uptake (8 μM final external concentration) in *A. baumannii* Δ3-pore cells carrying an empty vector, WT (AdeFGH), and AdeG efflux pump variants V141C, Q83A, S620A, and A705I. All cells were treated with SLUPP-1377 (0 μM-25 μM final external concentration) during kinetics. Each data point represent the average of two biological replictates with two technical repeats ± SD.

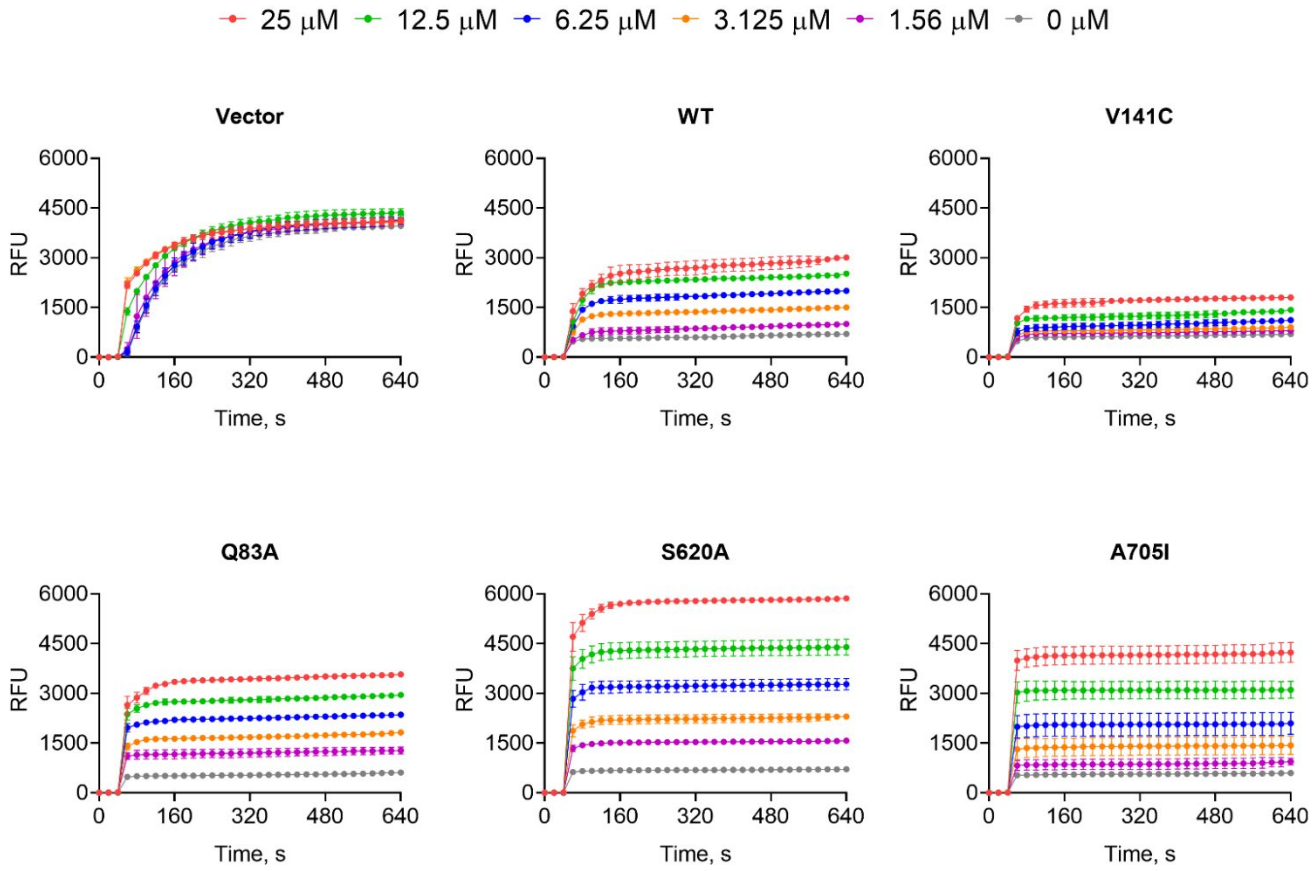

Figure S9. Kinetic curves of NPN uptake (8 μM final external concentration) in *A. baumannii* Δ3-pore cells carrying an empty vector, WT (AdeFGH), and AdeG efflux pump variants V141C, Q83A, S620A, and A705I. All cells were treated with SLUPP-1021 (0 μM-25 μM final external concentration) during kinetics. Each data point represent the average of two biological replictates with two technical repeats ± SD.

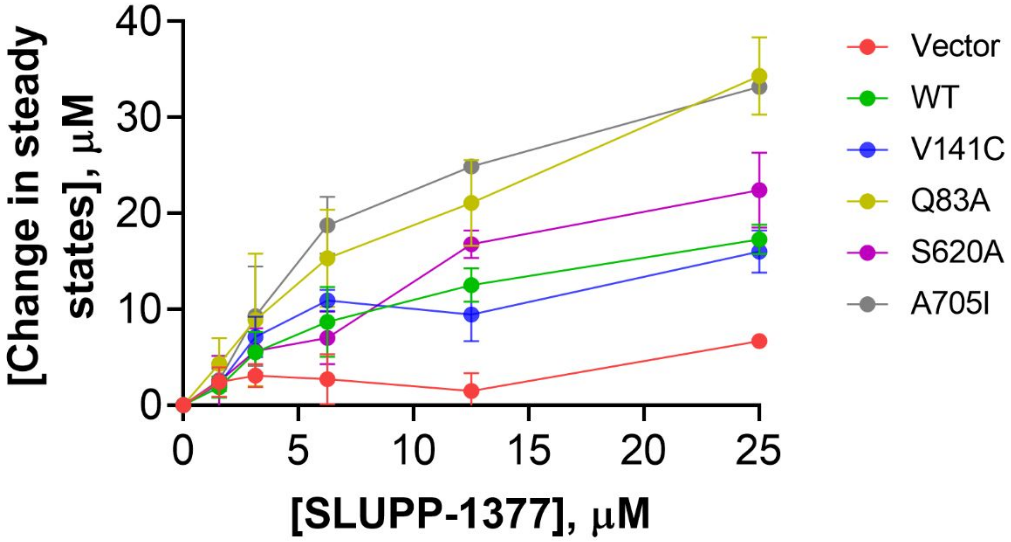

Figure S10. Steady-state EtBr (4 μM external concentration) accumulation levels calculated from the kinetic curves. SLUPP-1377 was used at the concentration ranging from 0 μM-25 μM. Each data point represents the average of two biological replicates with two technical repeats ± SD.

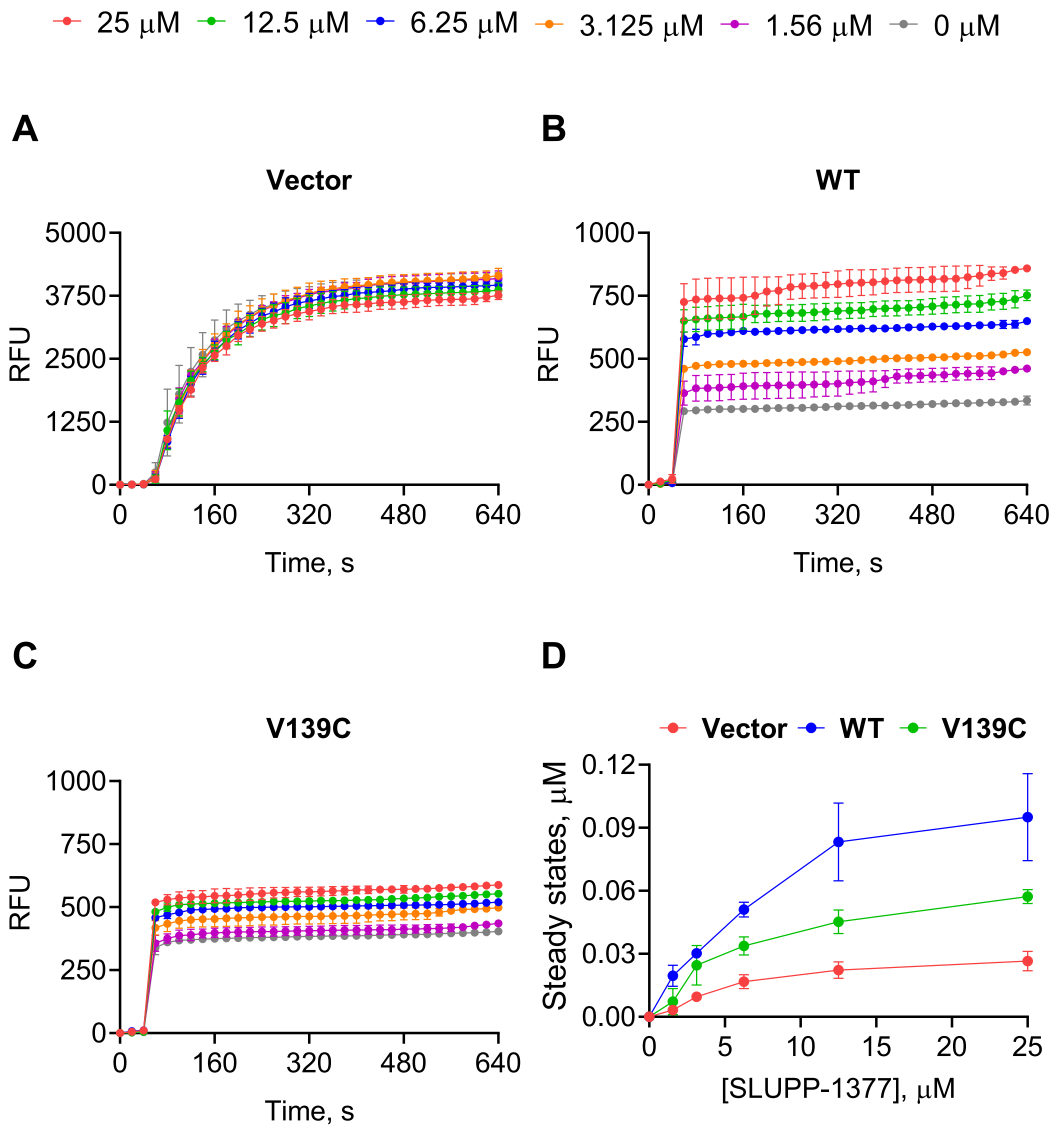

Figure S11. Kinetic curves and steady-state accumulation levels of NPN in *A. baumannii* Δ3-pore cells carrying an empty vector, WT (AdeIJK), and V139C (AdeJ) variant. (A-C) RFUs of NPN accumulation in the presence and absence of SLUPP-1377 and at the constant final NPN concentration of 8 µM. (D) Steady-state NPN accumulation levels calculated from the kinetic curves shown in A-C. Each data point represent the average of two biological replictates with two technical repeats ± SD.

Table S1. Quantitation of relative intensities in immunoblotting analysis of AdeG expression from efflux-deficient AYE Δ2 and Ab5075 Δ2 cells and their variants.

Strains	% Intensity
AYE Δ2	100
AYE Δ2 AdeL^{T319K}	368.77
Ab5075 Δ2	100
Ab5075 Δ2 AdeL^{V139G}	404.29
Ab5075 Δ2 AdeL^{IS5}	397.21

Table S2. MICs of substrate antibiotics and minimum potentiation concentration (MPC_4) of efflux pump inhibitors in the hyperporinated efflux-deficient *A. baumannii* ATCC17978 strain (vector) and the efflux-deficient strains producing the WT (AdeIJK) efflux pump and indicated AdeJ variants.

Strains	MICs				CHL MPC_4 (μM)		NOR MPC_4 (μM)	
	CHL (μg/mL)	NOR (μg/mL)	SLUPP-1377 (μM)	SLUPP-1021 (μM)	SLUPP-1377 (μM)	SLUPP-1021 (μM)	SLUPP-1377 (μM)	SLUPP-1021 (μM)
Vector	8	0.125	6.25	25	6.25	25	6.25	25
WT	32	4	100	>200	25	50	25	50
E675A	16	4	100	>200	25	50	25	50
N81A	32	8	100	>200	25	50	25	50
R701A	32	8	100	>200	25	50	25	25
G721I	16	4	100	>200	25	50	25	50
F618A	64	8	100	>200	12.5	25	12.5	25
A134I	32	4	100	>200	25	50	25	50
F136A	64	8	100	>200	12.5	50	12.5	25
V139C	32	4	100	>200	**100**	**100**	**100**	**100**
F178C	8	1	50	>200	**6.25**	**12.5**	**6.25**	**12.5**

Table S3. Fractional inhibitory concentration index (FICI) of naphthyl-substituted DAQs with substrate antibiotics in efflux-deficient *A. baumannii* AYE Δ2, Ab5075 Δ2, and ATCC17978 Δ3 and their corresponding AdeG overproducing variants

Strains	FICI					
	SLUPP-1377			SLUPP-1021		
	CHL	NOR	NAL	CHL	NAR	NAL
AYEΔ2	0.375	0.375	0.375	0.5	0.5	0.5
AYE Δ2 AdeL^{T319K}	0.3125	0.3125	0.375	0.3125	0.3125	0.375
Ab5075Δ2	0.5	0.5	0.5	0.5	0.5	0.5
Ab5075Δ2 AdeL^{V139G}	0.375	0.3125	0.375	0.375	0.125	0.5
Ab5075Δ2 AdeL^{IS5}	0.3125	0.281	0.3125	0.375	0.3125	0.375
Δ3 (Vector)	1.25	1.25	1.25	1.25	1.25	1.25
Δ3 (pAdeFGH) (WT)	0.5	0.375	0.375	0.75	0.3125	0.375

FICI - Synergy, ≤0.5; additivity, >0.5 - ≤1; no interaction (indifference), >1 - ≤4

Table S4. Quantitation of relative intensities in immunoblotting analysis of AdeG expression from *A. baumannii* ATCC17978 efflux-deficient AbΔ3 cells carrying an empty vector and the indicated plasmid-borne AdeG variants and corresponding hyperporinated derivatives.

Strains	% Intensity	
	Pore (-)	Pore (+)
Vector	-	-
WT	100	100
I621A	100.59	102.36
S620A	98.44	100.4
L138A	102.26	105.7
G679I	103.53	104.91
Y725A	101.66	99.98
A705I	102.91	97.76
Q83A	99.47	95.54
V141C	105.12	103.39
F180C	105.09	101.23
P136A	104	106.12

Table S5. Minimal inhibitory concentrations (MICs) of substrate antibiotics in the hyperporinated efflux-deficient *A. baumannii* ATCC17978 strain (vector) and the efflux-deficient strains producing the WT (AdeFGH) efflux pump and indicated AdeG variants.

Strains	Location	MICs (μg/mL)		
		CHL	NOR	NAL
Vector	-	8	0.25	2
WT	-	32	1	8
G679I	F-loop	8	0.25	2
Q83A	PBP	32	1	8
A705I		32	2	8
Y725A		16	0.5	4
S620A	G-loop	16	1	8
I621A		16	0.25	4
P136A	DBP	16	0.25	2
L138A		32	1	8
V141C		32	1	8
F180C		8	0.25	2

Abbreviations- CHL, Chloramphenicol; NOR, Norfloxacin; NAL, Nalidixic Acid

Table S6. Comparisons of the impact of single amino acid substitutions in the multidrug binding sites of AdeG and AdeJ on the inhibition abilities of EPIs.

Location	AdeG					AdeJ				
	Residues	SLUPP-1377 (MIC/MPC_4)		SLUPP-1021 (MIC/MPC_4)		Residues	SLUPP-1377 (MIC/MPC_4)		SLUPP-1021 (MIC/MPC_4)	
		CHL	NOR	CHL	NOR		CHL	NOR	CHL	NOR
-	Vector	1	1	1	1	Vector	1	1	1	1
-	WT	4	8	4	16	WT	4	4	8	8
F-loop	G679I	**1**	**2**	**1**	**2**	E675A	4	4	8	8
PBP	Q83A	8	16	8	32	N81A	4	4	8	8
	A705I	4	8	4	16	R701A	4	4	8	8
	Y725A	2	4	2	**4**	G721I	4	4	8	8
G-loop	S620A	4	**32**	4	**32**	-	-	-	-	-
	I621A	2	4	2	**4**	F618A	8	8	16	16
DBP	P136A	**2**	**2**	**2**	**2**	A134I	4	4	8	8
	L138A	8	16	8	16	F136A	8	8	8	16
	V141C	**1**	**1**	**1**	**2**	V139C	**1**	**1**	**4**	**4**
	F180C	**1**	**2**	**1**	**2**	F178C	8	8	**32**	**32**

Table S7. Comparisons of the impact of single amino acid substitutions in the multidrug binding sites on the transport and inhibition abilities of the efflux-pump specific substrates and EPIs, respectively.

Location	Antibiotics/Substrates			EPIs	
	AdeB	AdeG	AdeJ	AdeG	AdeJ
F-loop	I663C	-	-	-	-
	D664C	-	-	-	-
	E665A	G679I	E675A	G679I	E675A
PBP	-	Q83A	N81A	Q83A	N81A
	-	A705I	R701A	A705I	R701A
	W708C	-	-	-	-
		Y725A	G721I	Y725A	G721I
G-loop	W610C	-	-	-	-
	-	S620A	-	S620A	-
	-	I621A	F618A	I621A	F618A
DBP	E89A	-	-	-	-
	-	P136A	A134I	P136A	A134I
	-	L138A	F136A	L138A	F136A
	-	V141C	V139C	V141C	V139C
	F178C	F180C	F178C	F180C	F178C
	F277C	-	-	-	-
	W568C	-	-	-	-

Note: For antibiotics/substrates, green and pink colors represent partial loss and gain of function respectively. Blue represents similar activity compared to the wild-type pump. For EPIs, green and pink colors represent resistance and hypersusceptibility. Blue represents similar inhibition compared to the wild-type pump. The efflux and inhibition activities are substrate and inhibitor specific.

Antibiotics used for comparison in this table: AdeB-GEN[1], AdeG-NOR (this study), and AdeJ-NOV[2]

References

(1) Leus, I. V.; Roberts, S. R.; Trinh, A.; W. Yu, E.; Zgurskaya, H. I. Nonadditive functional interactions between ligand-binding sites of the multidrug efflux pump AdeB from Acinetobacter baumannii. *Journal of Bacteriology* **2024**, *206* (1), e00217-00223.
(2) Saral Sariyer, A.; Leus, I. V.; Tambat, R.; Farjana, M.; Olvera, M.; Rukmani, S. J.; Sariyer, E.; Smith, J. C.; Parks, J. M.; Walker, J. K.; et al. Mutations in the proximal binding site and F-loop of AdeJ confer resistance to efflux pump inhibitors. *Antimicrob Agents Chemother* **2025**, e0009025. DOI: 10.1128/aac.00090-25 From NLM Publisher.
